# Concurrent Targeting of HDAC and PI3K to Overcome Phenotypic Heterogeneity of Castration-resistant and Neuroendocrine Prostate Cancers

**DOI:** 10.1158/2767-9764.CRC-23-0250

**Published:** 2023-11-20

**Authors:** Ailin Zhang, Nathan A. Lau, Alicia Wong, Lisha G. Brown, Ilsa M. Coleman, Navonil De Sarkar, Dapei Li, Diana C. DeLucia, Mark P. Labrecque, Holly M. Nguyen, Jennifer L. Conner, Ruth F. Dumpit, Lawrence D. True, Daniel W. Lin, Eva Corey, Joshi J. Alumkal, Peter S. Nelson, Colm Morrissey, John K. Lee

**Affiliations:** 1 Division of Human Biology, Fred Hutchinson Cancer Center, Seattle, Washington.; 2 Department of Urology, University of Washington School of Medicine, Seattle, Washington.; 3 Department of Pathology, Medical College of Wisconsin, Milwaukee, Wisconsin.; 4 Department of Medicine, University of Washington School of Medicine, Seattle, Washington.; 5 Department of Laboratory Medicine and Pathology, University of Washington School of Medicine, Seattle, Washington.; 6 Division of Public Health Sciences, Fred Hutchinson Cancer Center, Seattle, Washington.; 7 Department of Internal Medicine, Rogel Cancer Center, University of Michigan, Ann Arbor, Michigan.; 8 Division of Clinical Research, Fred Hutchinson Cancer Center, Seattle, Washington.

## Abstract

Castration-resistant prostate cancer (CRPC) consists of multiple phenotypic subtypes including androgen receptor (AR)-active prostate cancer (ARPC) and neuroendocrine prostate cancer (NEPC). Tumor cells with these phenotypes can coexist between metastases within a patient and within an individual tumor. Treatments that are effective across CRPC subtypes are currently lacking. Histone deacetylation is crucial for the regulation of chromatin structure and maintenance of cancer cell state and activation of the PI3K/AKT/mTOR signaling cascade is a tumor growth–promoting pathway. We therefore investigated combined targeting of histone deacetylase (HDAC) and PI3K using a rationally designed dual inhibitor, fimepinostat, in CRPC subtypes *in vitro* and *in vivo*. Dual HDAC1/2 and PI3K/AKT pathway inhibition by fimepinostat led to robust tumor growth inhibition in both ARPC and NEPC models including cell line– and patient-derived xenografts. HDAC1/2 inhibition combined with PI3K/AKT inhibition was more effective than targeting each pathway alone, producing growth inhibitory effects through cell-cycle inhibition and apoptosis. Molecular profiling revealed on-target effects of combined HDAC1/2 and PI3K/AKT inhibition independent of tumor phenotype. Fimepinostat therapy was also associated with the suppression of lineage transcription factors including AR in ARPC and Achaete-scute homolog 1 (ASCL1) in NEPC. Together, these results indicate that fimepinostat represents a novel therapeutic that may be effective against both ARPC and NEPC through CRPC subtype-dependent and -independent mechanisms.

**Significance:**

CRPC is a heterogeneous disease constituting multiple phenotypic subtypes that often co-occur within tumors or across metastases in patients. Existing targeted therapies for CRPC do not take this into account. Here we show that fimepinostat, a dual HDAC1/2 and PI3K/AKT inhibitor investigated clinically in other cancer types but not prostate cancer, may overcome this heterogeneity by effectively inhibiting both ARPC and NEPC subtypes of CRPC.

## Introduction

When initially diagnosed, the vast majority of metastatic prostate cancers exhibit a phenotype consistent with a secretory luminal cell that is regulated by androgens through the androgen receptor (AR) signaling program. Inhibiting the AR signaling pathway through androgen deprivation therapy (ADT) produces exceptional clinical responses in the majority of patients. However, resistance to ADT is universal, leading to a clinical state termed castration-resistant prostate cancer (CRPC; refs. 1–3). CRPC consists of multiple molecular and histologic subtypes of prostate cancer including neuroendocrine prostate cancer (NEPC) which takes on characteristics of neuroendocrine (NE) differentiation and is no longer responsive to AR-directed treatments. Furthermore, detailed studies of metastatic tumors have demonstrated the existence of both intertumor and intratumor heterogeneity with respect to the coexistence of ARPC and NEPC phenotypes within patients. Consequently, developing effective therapeutics strategies will require approaches that simultaneously cotarget divergent lineages and phenotypes (4). Histone deacetylation is crucial for regulating chromatin structure, gene transcription, and the epigenetic state of the cell. Drugs targeting epigenomic structures enforced by strong lineage drivers, such as the AR in ARPC and Achaete-scute homolog 1 (ASCL1) in NEPC, have the potential to disrupt cell survival programs and induce growth arrest. To date, enzymes that regulate histone acetylation have been developed, though none are clinically approved for cancer treatment. In addition, NEPC can be highly aggressive and highly proliferative. This would suggest that a combination therapy focused on disrupting both the tumor phenotype and the proliferative aspect of the tumor may be more effective. The PI3K/AKT/mTOR pathway is a well-established tumor growth–promoting pathway in ARPC (5, 6). Recent clinical trials, targeting CRPC with abiraterone and ipatasertib (an AKT inhibitor) showed superior antitumor activity to abiraterone alone, especially in patients with PTEN-loss tumors (7, 8).

In this study, we sought to determine whether using clinically available drugs targeting both histone deacetylase (HDAC) and PI3K/AKT activities can exploit these vulnerabilities in treatment-resistant AR-positive CRPC and AR-negative NEPC tumor phenotypes. We evaluated romidepsin an FDA-approved HDAC1/2 inhibitor used in the treatment of T-cell lymphoma, and ipatasertib, an AKT inhibitor that has shown preliminary efficacy in clinical trials in patients with CRPC (7, 8). While these inhibitors have been tested in clinical trials, they have not been used in combination to treat CRPC previously. We also tested fimepinostat, a dual HDAC and PI3K inhibitor that has produced prolonged treatment responses in a phase II clinical trial in patients with diffuse large and high-grade B-cell lymphomas (9). We determined that dual inhibition of HDACs and the PI3K/AKT pathway is highly effective in suppressing both ARPC and NEPC tumor growth, and demonstrated synergy in several CRPC models. Our findings support cotargeting HDAC1/2 and PI3K/AKT as an attractive therapeutic strategy for advanced CRPC where intertumor and intratumor heterogeneity contribute to treatment resistance.

## Materials and Methods

### Cell Lines and Tumor Models

All cells were maintained at 37°C in humidified Steri-Cult CO_2_ incubators (Thermo Fisher Scientific). C4-2B (ATCC, CRL-3314, RRID:CVCL_4784), 22Rv1 cells (ATCC, CRL-2505, RRID:CVCL_1045), VCaP (ATCC, CRL-2876, RRID:CVCL_2235), LNCaP (ATCC, CRL-1740, RRID:CVCL_0395), LNCaP95 (gift from Stephen Plymate, University of Washington (Seattle, WA), RRID:CVCL_ZC87), LAPC4 (gift from Robert Reiter, UCLA, RRID:CVCL_4744), PC3 (ATCC, CRL-1435, RRID:CVCL_0035), and DU145 (ATCC, HTB-81, RRID:CVCL_0105) cell lines were maintained in RPMI1640 Media or DMEM (Gibco, Life Technologies) with 10% FBS (Atlanta Biologicals) or 10% charcoal-stripped FBS (Thermo Fisher Scientific). RWPE-1 (ATCC, CRL-11609, RRID:CVCL_3791) and HPrEC (ATCC, PCS-440-010) were cultured in Keratinocyte Serum Free Medium (Thermo Fisher Scientific) supplemented with 0.05 mg/mL bovine pituitary extract and 5 ng/mL human epidermal growth factor. NCI-H660 (ATCC, CRL-5813, RRID:CVCL_1576), MSKCC EF1 (gift from Yu Chen, Memorial Sloan Kettering Cancer Center), and LASCPC-01 (ATCC, CL-3356, RRID:CVCL_UE17) cell lines were maintained in HITES media (ATCC) with 5% FBS (Atlanta Biologicals). Short tandem repeat (STR) profiling of cell lines was performed on a yearly basis and *Mycoplasma* testing was performed twice yearly. To further expand NCI-H660 cell numbers, NCI-H660 cells were injected subcutaneously in immune compromised male mice as described previously (10) and tumors harvested and dissociated using the human Tumor Dissociation Kit (Miltenyi Biotec) according to manufacturer's protocol and used for *in vitro* experiments. Similarly, LuCaP patient-derived xenograft (PDX) tumors [established in-house (10)], were harvested and dissociated using the human Tumor Dissociation Kit (Miltenyi Biotec) according to manufacturer's protocol. Cell lines stably engineered for doxycycline-inducible short hairpin RNA (shRNA) expression have been described previously (11). The CWR-R1-D567 cell line (gift from Stephen Plymate, University of Washington, Seattle, WA, RRID:CVCL_ZC61f) genomically engineered using TAL effector nuclease to delete exons 5–7 of the AR gene has been described previously (12). All cell lines used in this project were validated through STR analysis using ATCC reference genomes.

### Viability Assays

For dose–response experiments, 22Rv1 (5 × 10^3^), LNCaP (5 × 10^3^), LNCaP95 (5 × 10^3^), LAPC4 (5 × 10^3^), VCaP (1 × 10^4^), MSKCC EF1 (1 × 10^4^), NCI-H660 (1 × 10^4^), RWPE-1 (1 × 10^4^), HPrEC (1 × 10^4^), DU145 (2.5 × 10^3^), and PC3 (2.5 × 10^3^) were seeded in 96-well flat-bottom, white wall plates (Corning). Cells were treated with 3-fold serial dilutions of fimepinostat with a starting dose of 1 µmol/L with eight replicates of each condition at 37°C for 96 hours. Genetically modified 22Rv1 cells engineered to express GFP, AR-FL, c-Myc, or myristoylated AKT1 (myrAKT1) were treated similarly. Cell viability was determined using the CellTiter-Glo 2.0 Assay (Promega). Studies comparing fimepinostat to vorinostat (Sigma) and quisinostat (Selleck) were conducted in a similar fashion except the starting concentration for vorinostat was 10 µmol/L.

LuCaP PDX cells and NCI-H660 cells (2 × 10^4^), 22Rv1 (5 × 10^3^), and C4-2B cells (5 × 10^3^) were seeded in 96-well plates in either phenol red–free RPMI1640 media (Gibco) supplemented with 5% charcoal/dextran-treated FBS (Atlanta Biologicals) and 1X pen/strep (Gibco) or HITES media (ATCC). Cells were treated 4–6 hours after seeding with vehicle, romidepsin, BYL719, AZD8186, ipatasertib, vorinostat, entinostat, mocetinostat, fimepinostat (Medchemexpress), or quisinostat (Selleck) in three replicate wells. Viability was determined at time 0, 48, 72, or 96 hours after treatment depending on the assay requirements, using the CellTiter Glo Viability Assay (Promega) according to manufacturer's protocols.

### Cell-cycle Analysis by Flow Cytometry and Caspase Assays

22Rv1 and LNCaP95 cells were treated with fimepinostat 1 µmol/L for 24 hours. In addition, C4-2B and NCI-H660 cells were treated with a range of concentrations of romidepsin, ipatasertib, BYL719, AZD8186, fimepinostat, and vehicle control for 48 hours. To fix the cells, 2 × 10^6^ cells from each condition were resuspended in 300 µL of PBS and 700 µL of ice-cold 100% ethanol was added dropwise while continuously vortexing the solution. Fixed cells were incubated at 4°C overnight. Prior to flow cytometry analysis, cells were washed in PBS to remove residual ethanol and resuspended in 500 µL of PBS with 50 µg/mL propidium iodide (Thermo Fisher Scientific) and 0.5 µg/mL of RNase A (Thermo Fisher Scientific). Cells were incubated at 37°C for 30 minutes before flow cytometry analysis on a SH800S Cell Sorter (Sony).

For the apoptosis assay, 2.5 × 10^5^ C4-2B and 7.5 × 10^5^ NCI-H660 cells were seeded in 96-well plates and treated with a range of concentrations of romidepsin, ipatasertib, BYL719, AZD8186, fimepinostat, and vehicle control for 48 hours. Apoptosis was assessed using an ApoTox-Glo Triplex Assay System (Promega) measuring caspase 3/7 activity as per the manufacturer's instructions and luminescence measured on a Varioskan Lux (Thermo Fisher Scientific).

### *In Vivo* Testing

All animal procedures used in this study were approved by the Institutional Animal Care and Use Committee at the University of Washington (Seattle, EA; Protocol Number: 3202-01) and the Fred Hutchinson Cancer Center in accordance with the NIH guidelines. The LuCaP 35CR (adenocarcinoma), and LuCaP 145.1 and LuCaP 208.1 (NE) PDX models were maintained by serial passaging in CB-17, SCID male mice as described previously (10). CB-17 SCID mice (Charles River Laboratories) were implanted subcutaneously with either 2 × 10^6^ 22Rv1 cells or LuCaP 35CR, 145.1, or 208.1 tumor tissue. Animals underwent rolling enrollment once tumors exceeded 100 mm^3^ and were randomized into two groups. For the romidepsin studies: Group 1 received control vehicle (PBS), intraperitoneally, 200 µL, two times weekly for up to 3.5 weeks. Group 2 received 1.5 mg/kg romidepsin in vehicle, intraperitoneally, 200 µL, two times weekly for up to 3.5 weeks. For the fimepinostat study: Group 1 received control vehicle (captisol, 30% w/v), orally, 200 µL, five times weekly for up to 3.5 weeks. Group 2 received 75 mg/kg fimepinostat in vehicle, orally, 200 µL, five times weekly for up to 3.5 weeks. Two mice per group were euthanized at 4 days after enrollment for early timepoint analysis, and the remaining 7–9 mice per group were dosed for up to 3.5 weeks. Tumor volumes (TV) were measured twice weekly using Fowler Ultra-Cal calipers (calculated as *L* × *H* × *W* × 0.5236). TVs and body weights (BW) were collected twice weekly. In addition, animals were monitored at least three times weekly for health conditions and abnormal behaviors associated with pain and distress. Animals were euthanized after the 3.5 week dosing period, if TVs exceeded 1,000 mm^3^, if BW fell below 20%, or if animals showed other signs of health compromise (i.e., body condition score < 2, ulcerating tumors, lethargy, piloerection). All efforts were made to minimize suffering and no unexpected mortality occurred outside of planned euthanasia or humane endpoints. For euthanasia, mice were anesthetized by an intraperitoneal injection of a ketamine/xylazine (130 mg/8.8 mg/kg) cocktail and bled by cardiac puncture immediately followed by cervical dislocation or alternatively placed in a CO_2_ chamber until evident signs of narcosis followed by cervical dislocation. At sacrifice, the tumors were divided equally into paraffin blocks and flash frozen for subsequent molecular analyses.

### *Ex Vivo* Tissue Slice Culture Testing

Fresh xenograft tumors were harvested and collected in RPMI1640 supplement with 100 U/mL penicillin, 100 µg/mL streptomycin, and 2.5 µg/mL amphotericin B. Tumors were sliced to generate 330 µm tissue slices using a Krumdieck Tissue Slicer (Alabama Research and Development). Tissue slices were placed on BioCoat Control Inserts (Corning) which were inserted into 6-well plates containing tissue slice culture medium (13). To ensure a dynamic air-liquid interface on the tissue slices, the plates were placed on an orbital shaker at 15 rpm during incubation at 37^o^C. Tissues slices were equilibrated in culture for 24 hours prior to drug treatment. Tissue slices were treated with DMSO or fimepinostat (30, 100, or 300 nmol/L) for 3 days. To account for tissue slice and tumor heterogeneity, at least six slices were cultivated for each condition. Tissue slice viability analysis was performed using the Realtime-Glo MT Cell Viability Assay (Promega) according to the manufacturer's protocol.

### RNA Sequencing and Analysis

RNA was isolated from *in vitro* cultures of dissociated LuCaP tumors (LuCaP 35CR, 49, 93, 136CR, 145.1, 173.1, 208.1) and C4-2B, 22Rv1, and NCI-H660 cell lines treated with vehicle or romidepsin (500 pmol/L or 1 nmol/L concentration for 96 hours or short-term treatment for 24 hours). Alternatively, 22Rv1, LNCaP95, NCI-H660, and MSKCC EF1 cells were treated with either DMSO or fimepinostat (10 nmol/L, 100 nmol/L, or 1 µmol/L) for 24 hours before RNA isolation. C4-2B and NCI-H660 were also cell lines treated with vehicle, romidepsin (1 nmol/L), ipatasertib (1 µmol/L), a combination of romidepsin and ipatasertib (1 nmol/L and 1 µmol/L, respectively), or fimepinostat (25 nmol/L) for 24 hours using RNA STAT-60 (Tel-Test). The isolated RNA was then purified with Qiagen RNeasy Kit (Qiagen Inc.) according to the manufacturer's protocol utilizing the DNase treatment in solution prior to purification. RNA concentration, purity, and integrity were assessed by NanoDrop 2000 (Thermo Fisher Scientific Inc) and 2100 Bioanalyzer (Agilent Technologies). RNA sequencing (RNA-seq) libraries were constructed from 1 mg total RNA using the Illumina TruSeq Stranded mRNA LT Sample Prep Kit according to the manufacturer's protocol. Barcoded libraries were pooled and sequenced on a NovaSeq S1 100 flowcell generating 50 bp paired end reads. Sequencing reads were mapped to the STARv2.7.3a hg38 human (NCI-H660 also aligned to mm10; mouse subtracted with XenofilteR as they were derived from NCI-H660 xenografts; ref. 14). All subsequent analyses were performed in R. Gene-level abundance was quantitated from the filtered human alignments using Genomic Alignments (15). Differential expression was assessed using transcript abundances as inputs to the limma (16), filtered for a minimum expression level of at least 1 count per million reads in three samples prior to testing, using the Benjamin–Hochberg FDR adjustment. Gene expression results were ranked by their limma Voom statistics and used to conduct gene set enrichment analysis (GSEA) to determine patterns of pathway activity in treatment groups utilizing the pathways from within the MSigDBv7.5.1 (17). To investigate gene sets in common that were over-represented in fimepinostat and ipatasertib + romidepsin treated C4-2B and NCI-H660 cells relative to control, we used an over-representation enrichment tool http://www.gsea-msigdb.org/gsea/msigdb/annotate.jsp (17).

### AR and Myc Transcript and Protein Profiling Studies

To assess the effect of fimepinostat on AR and Myc transcripts, cells were seeded at 1 × 10^5^ cells/well in a 12-well plate and treated with DMSO or fimepinostat 1 µmol/L for 1, 2, 4, and 8 hours to examine AR transcripts and 0.25, 0.5, 1, 1.5, and 2 hours to examine Myc transcripts. To investigate mRNA stability, cells were treated with actinomycin D (Sigma) 1 µg/mL in addition to DMSO or fimepinostat. A total of 1 µg of total RNA was used for each reverse transcription reaction with SuperScript IV Reverse Transcriptase (Thermo Fisher Scientific). Real-time PCR reactions were set up in quadruplicate with 5 ng cDNA and TaqMan Gene Expression Assays (for AR: Hs00171172_m1, Hs04260217_m1, Hs04117242_cn_F; for c-Myc: Hs00153408_m1, Hs03660964_cn; for GAPDH: Hs99999905_m1) in 10 µL reactions and analyzed on a QuantStudio 5 System (Applied Biosystems). Relative quantification (RQ) was reported as fold change (2^ΔΔCt^ calculations) normalized to the 0 hour timepoint and the housekeeping gene GAPDH multiplexed within the same reaction as a reference.

To assess the time-dependent effect of fimepinostat on AR and Myc levels, 22Rv1 and LNCaP95 cells were plated and treated with fimepinostat for up to 24 hours before collection of protein lysates. To evaluate AR and Myc protein stability by cycloheximide chase assay, cells were plated and treated with 10 µg/mL cycloheximide in combination with DMSO or fimepinostat 1 µmol/L. Lysates were collected after treatment for 0, 1, 2, 4, 8, 16, and 24 hours to evaluate AR protein stability and 0, 0.25, 0.5, 1, 1.5, and 2 hours to evaluate Myc protein stability.

### Tissue Microarray Construction and Analysis

Each tumor was fixed in buffered formalin and embedded in paraffin where available. A tissue microarray (TMA) was constructed using duplicate 1-mm-diameter cores from vehicle control, romidepsin-, and fimepinostat-treated tumors. LuCaP 35CR, LuCaP 145.1, LuCaP 208.1, and 22Rv1 xenografts were constructed from viable tumor tissue from vehicle and treated tumors. To assess cell proliferation, dividing tumor cell nuclei were counted in four fields containing viable tumor cells at 200X magnification by two blinded observers (C. Morrissey and L.G. Brown). Unusable samples, including missing, or necrotic tissue cores, were excluded from final analysis.

### HDAC Activity Assay

The HDAC-Glo I/II activity assay (Promega) was used to measure HDAC activity as per the manufacturer's instructions. Briefly, cells [C4-2B, 22Rv1 (5 × 10^3^), NCI-H660, or LuCaP 173.1CL (1 × 10^4^)] were seeded in 96-well plates and treated with increasing concentrations of romidepsin or a control HDAC inhibitor (trichostatin A) for 1 hour. An equal volume of HDAC-Glo I/II reagent was added to the wells and luminescence measured on a GENios plate reader (Tecan).

### Immunoblot Analysis

Whole-cell protein extracts from LuCaP PDX models and cell lines were obtained using the Nuclear Extract Kit (Active Motif) according to manufacturer's protocols. Quantification of total protein was determined using the RC DC Protein Assay (Bio-Rad) or Pierce Rapid Gold BCA Protein Assay Kit (Thermo Fisher Scientific) according to manufacturer's protocols. Twenty to 30 µg of total protein lysate were electrophoresed on either 4%–15% Bis-Tris gels (Bio-Rad) with 1x Tris/Glycine/SDS Buffer (Bio-Rad) or Bolt 4%–12% Bis-Tris Plus gels (Thermo Fisher Scientific) with Novex Bolt MES SDS Running Buffer (Thermo Fisher Scientific). The proteins were transferred to nitrocellulose that was blocked with either 5% Blotting-Grade Blocker (Bio-Rad) in TBS/0.1% Tween-20 or 5% cow's milk in PBS/0.1% Tween-20 and subsequently probed with primary antibodies [Histone H3 (D1H2) XP Rabbit mAb #4499, 1:2,000; Acetyl-Histone H3 (Lys27) (D5E4) XP Rabbit mAb #8173, 1:1,000; ACTIN Sigma A2228, 1:2500; PARP-1 (F-2) Santa Cruz Biotechnology sc-8007, 1:1,000; Caspase-3 (8G10) Rabbit mAb #9665, 1:1,000; Cleaved Caspase-3 (Asp175) Ab #9661, 1:1,000; GAPDH (GT239) Genetex GTX627408, 1:10,000; AR (441) Santa Cruz Biotechnology sc-7305, 1:1,000; AR (ARv7 Splice Variant) Genetex GTX33604, 1:1,000; Glucocorticoid Receptor (GR; D6H2L) XP Rabbit mAb #12041, 1:1,000; c-Myc (D84C12) Rabbit mAb #5605, 1:1,000; Phospho-Akt (Ser473) (D9E) XP Rabbit mAb #4060, 1:1,000; Akt (pan) (C67E7) Rabbit mAb #4691, 1:1,000; Cyclin D3 (DCS22) Mouse mAb #2937, 1:1000; p21 Waf1/Cip1 (12D1) Rabbit mAb #2947, 1:1,000; Phospho-cdc2 (Tyr15) (10A11) Rabbit mAb #4539, 1:1,000; Cdc2 (POH1) Mouse mAb #9116, 1:1,000; Cyclin B1 Antibody #4138, 1:1,000; ASCL1 (D-7) Santa Cruz Biotechnology sc-374104, 1:1,000; Monoclonal Anti-FLAG M2-Peroxidase Ab Sigma A8592, 1:5,000; HA-Tag (C29F4) Rabbit mAb #3724, 1:1,000; Notch2 (D76A6) XP Rabbit mAb #5732, 1:1,000; Notch3 (D11B8) Rabbit mAb #5276, 1:1,000] and secondary antibodies. Proteins were visualized using Clarity Western enhanced chemiluminescence Substrate (Bio-Rad) or Immobilon Western Chemiluminescent horseradish peroxidase (HRP) Substrate (Millipore) and a ChemiDoc imaging system (Bio-Rad).

### IHC Analysis

For tissue slice culture, formalin-fixed and paraffin-embedded tissue sections on positively charged glass slides were incubated at 65°C for 1 hour, placed in xylene for 5 minutes to deparaffinize the sections, and rehydrated with successive washes for 5 minutes each in 100%, 95%, and 70% ethanol. Antigen retrieval was performed by boiling the slides in 0.2 mol/L citric acid and 0.2 mol/L sodium citrate in a pressure cooker. Tissue sections were washed with PBS, blocked with 2.5% horse serum for 1 hour, and incubated with primary antibody [Ki67 Ab Novus Biologicals NB110-89717, 1:100; Cleaved Caspase-3 (Asp175) Ab #9661, 1:400; AR (441) Santa Cruz Biotechnology sc-7305, 1:100; c-Myc Ab (Y69) Abcam ab32072, 1:100; ASCL1 antibody BD Biosciences B556604, 1:100] diluted in 2.5% horse serum for 12–18 hours at 4°C. Endogenous peroxidases were blocked with hydrogen peroxide. Tissue sections were washed with PBS and incubated with secondary ImmPRESS-HRP anti-mouse or anti-rabbit IgG antibodies (Vector Laboratories) for 1 hour at room temperature. Staining was visualized with DAB peroxidase substrate (Dako) and quenched in water. Tissue sections were counterstained with hematoxylin prior to dehydration with successive washes for 5 minutes in 70%, 95%, and 100% ethanol and xylene followed by mounting with a glass coverslip using VECTASHIELD Antifade Mounting media (Vector Laboratories). Digital slide images were captured on a VENTANA DP 200 slide scanner (Roche) and quantification of average nuclear density was performed using QuPath-0.2.3 and ImageJ Fiji (RRID:SCR_003070; ref. 18).

For patient tissue analysis, automated IHC was performed on metastatic sites from patients with CRPC who enrolled in the rapid autopsy program at the University of Washington (Seattle, WA; Institutional Review Board number: 2341) on the VENTANA Discovery Ultra (Ventana Medical Systems Inc.) autostainer. Onboard deparaffinization was conducted in DISCOVERY Wash buffer (VMSI, 950-510). Subsequent heat-induced epitope retrieval was performed in DISCOVERY CC1 solution (VMSI, 950-500). TMA sections were incubated with AR monoclonal rabbit antibody (Cell Signaling Technology, Clone D6F11, catalog no. 5153S) and synaptophysin rabbit monoclonal antibody (Epredia Clone SP11, catalog no. RM-9111-S, RRID:AB_149938) at a dilution of 1:100 and 1:50, respectively in Ventana Antibody Diluent with Casein (VMSI, 760-219). The primary antibody was bound with DISCOVERY anti-Rabbit HQ secondary antibody (VMSI, 760-4815, RRID:AB_2811171), followed by DISCOVERY Anti-HQ HRP (VMSI, 760-4820) enzyme conjugate. To further increase signal intensity, tyramide amplification was achieved with the DISCOVERY HQ Amp kit (VMSI, 760-052) followed by DISCOVERY Amp Anti-HQ HRP (VMSI, 760-4602). The antibody complex was visualized using the ChromoMap DAB detection Kit (VMSI, catalog no. 760-159). Hematoxylin II (VMSI, 790-2208) and Bluing Reagent (VMSI, 760-2037) were used to counterstain the sections. Slides were scanned at a 40X magnification using the Ventana DP 200 instrument (VMSI) and visualized with HALO (Indica Labs).

### Genomic Analysis of Cell Lines and PDX Models

#### PDX Genome Analysis

Paired-end whole-exome and whole-genome next-generation sequencing was performed using Illumina HiSeq 2500 on genomic DNA isolated from PDX tissues. Paired germline genomic DNA was isolated from PDX tissue donor's blood. Sequence reads were aligned to the human reference genome hg38 using the Burrows-Wheeler Aligner-mem tool (BWA, RRID:SCR_010910; ref. 19). GATK best practices recommendation was adopted for processing all aligned sequence files (20). Germline and somatic mutation analyses were performed using Haplotype Caller and Mutect2. All high quality called mutations were annotated using ANNOVAR hg38 (21). Copy number was derived following the standardized Sequenza pipeline (22) requires tumor and normal paired sequencing data to call genome-wide copy-number analysis. The structural aberrations were determined using Pindell and SBAVA (23, 24). All mutations and genomic aberrations in selected gene sets were manually curated for accuracy and functional significance were reevaluated through database search and curation. We retrieved ploidy estimates from the tumor-normal paired copy-number analysis as inferred from curated Sequenza solutions.

#### Cell Line Genome Analysis

22Rv1 and C4-2B cell line exome sequence data are analyzed by adopting tumor-only pipeline of calling mutation and copy-number determination following standard BWA alignment to HG38 reference genome and GATK good practices steps. A tumor-only analysis is unfavorable for accurate ploidy estimation, so we avoided reporting a ploidy estimates of these cell lines. Because the NCI-H660 exome sequence data were unavailable, we retrieved the desired genes’ genotype calls (*RB1*, *PTEN*, *AKT1*, *PIK3CA*, *PIK3R1*) from public data portals (DepMap portal).

### Statistical Analysis

TV and BW between vehicle control and treated animals were compared when >2 animals remained in each group. Differences in TV and BW between vehicle control and treated animals were calculated using unpaired *t* tests with significance set at *P* ≤ 0.05. For cell division and IHC staining comparisons, unpaired *t* tests with unequal variances and significance set at *P* ≤ 0.05 were utilized. Statistical significance cutoffs are listed in figure legends. The SynergyFinder web application (https://synergyfinder.org/#!/) was used to determine the Loewe synergy score (25).

### Data Availability Statement

RNA-sequencing data for the LuCaP PDX biospecimens can be retrieved at Gene Expression Omnibus (RRID:SCR_005012) through accession number GSE223758. All other data and material will be made available upon request.

## Results

### Fimepinostat Therapy Demonstrates Broad Growth Inhibitory Effects Across Human Prostate Cancers with Diverse Phenotypes

**FIGURE 1:**
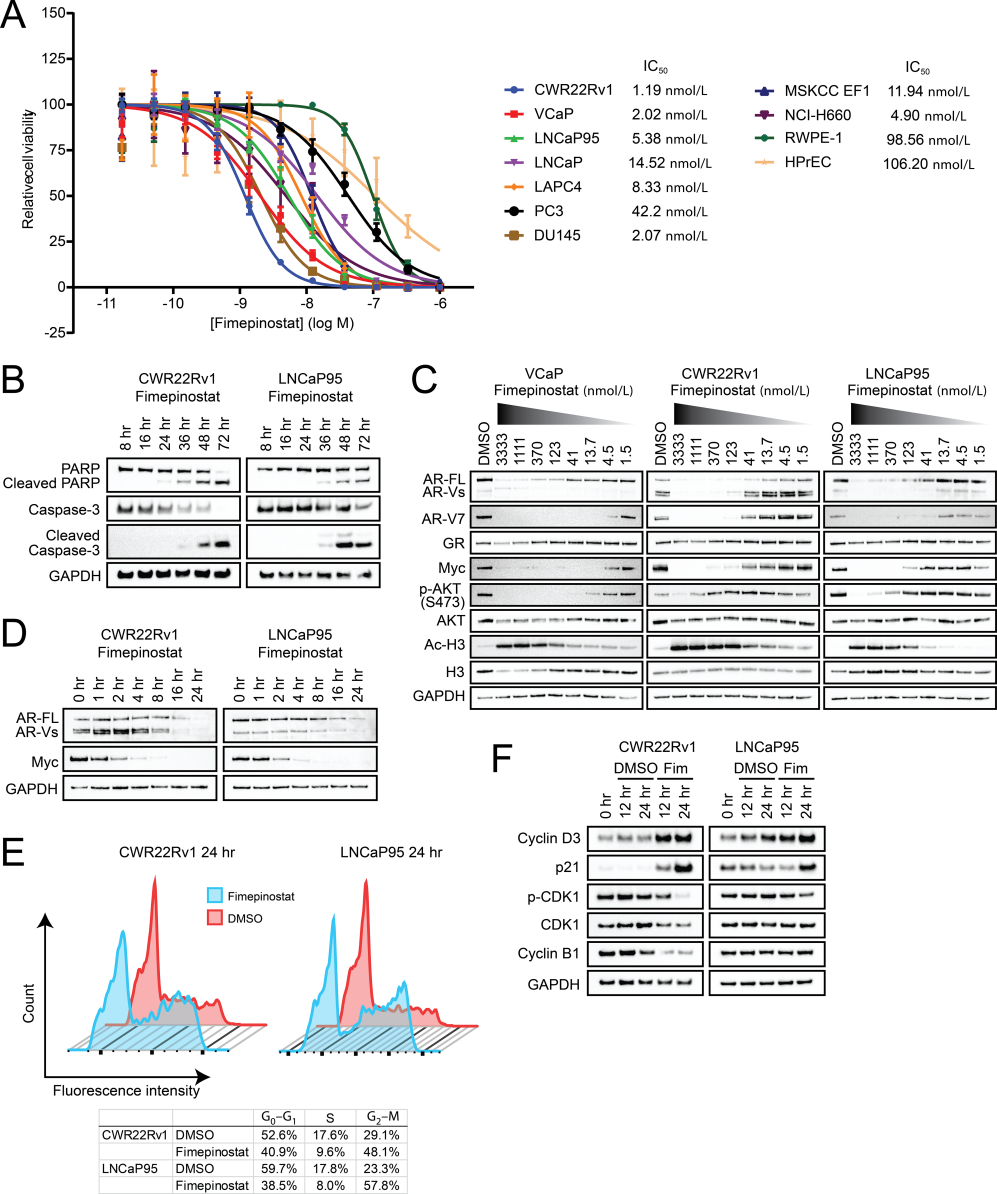
Fimepinostat demonstrates broad antiproliferative activity across prostate cancer cell line models and is associated with downregulation of AR and Myc expression and G_2_–M cell-cycle arrest. **A,** Fimepinostat dose–response curves for prostate cancer (AR^+^: 22Rv1, VCaP, LNCaP95, LNCaP, and LAPC4; AR^−^/NE^−^: DU145 and PC3; NE^+^: MSKCC EF1 and NCI-H660) and benign prostate epithelial (RWPE-1 and HPrEC) cell lines treated for 96 hours with cell viability normalized to DMSO control. Immunoblot analyses of the 22Rv1 and LNCaP95 cell lines treated with 0.1% DMSO or 1 µmol/L fimepinostat over a time course to assess markers of apoptotic cell death (**B**); the VCaP, 22Rv1, and LNCaP95 cell lines after 24 hours of treatment with DMSO control or increasing doses of fimepinostat to evaluate for on-target activity (Ac-H3) and effects on AR, GR, Myc, and AKT (**C**); and the 22Rv1 and LNCaP95 cell lines treated with 1 µmol/L fimepinostat over a time course to investigate the kinetics of effects on AR and Myc expression (**D**). **E,** DNA cell-cycle analysis plots of 22Rv1 and LNCaP95 cells after 24 hours of treatment with 0.1% DMSO (red) or 1 µmol/L fimepinostat (blot). **F,** Immunoblot analyses of the 22Rv1 and LNCaP95 cell lines treated with 0.1% DMSO or 1 µmol/L fimepinostat over a time course to determine the expression and activity of cell-cycle regulators.

We and others have demonstrated that metastatic CRPC (mCRPC) exhibit a substantial degree of heterogeneity, with tumors comprising ARPC features coexisting in the same patient with tumors exhibiting NEPC characteristics (Supplementary Fig. S1; ref. 2). To test the hypothesis that combinatorial repression of HDAC1/2 and PI3K/AKT signaling would effectively inhibit the growth of both ARPC and NEPC phenotypes, we determined dose–response effects of the dual HDAC/PI3K inhibitor fimepinostat on the growth and cell viability of a broad panel of human prostate cancer and benign prostate epithelial cell lines. Prostate cancer cell lines, including those lacking AR expression (PC3 and DU145) and those with NE phenotype (MSKCC EF1, NCI-H660), demonstrated IC50s that ranged from 0.82 to 42.2 nmol/L (mean 9.3 nmol/L; Fig. 1A). In contrast, the benign prostate epithelial cell lines were less sensitive to fimepinostat with IC_50_s of approximately 100 nmol/L, indicating preferential antiproliferative activity in the malignant state. We examined the temporal onset of markers of cell death in the 22Rv1 and LNCaP95 prostate cancer cell lines upon treatment with fimepinostat. Immunoblot analyses showed clear evidence of cleaved PARP and cleaved caspase-3 as early as 36 hours after treatment initiation (Fig. 1B).

HDACs are established as being required for AR function in prostate cancer and HDAC inhibition can lower AR protein levels by suppressing AR mRNA transcription (26). Furthermore, fimepinostat has previously been shown to downregulate Myc and suppress the growth of Myc-dependent cancers (27). We therefore examined dose-dependent effects of fimepinostat treatment on these and other targets in the VCaP, 22Rv1, and LNCaP95 cell lines. In these lines, HDAC inhibition occurred at low nanomolar doses based on hyperacetylation of histone H3 while PI3K/AKT pathway inhibition was evident at either low nanomolar (VCaP) or high nanomolar doses (22Rv1, LNCaP95) based on phosphorylation of AKT at Ser473 (Fig. 1C). Fimepinostat treatment suppressed the expression of both full-length AR (AR-FL) and AR splice variants (AR-Vs) including AR-V7 but did not affect the GR, another nuclear hormone receptor linked to resistance to AR targeting (ref. 28; Fig. 1C). Importantly, reduced AR and Myc protein expression occurred at dose levels that correlated more with HDAC inhibition than PI3K/AKT pathway inhibition.

To further investigate whether these effects on AR and Myc levels may be associated with HDAC inhibition, we tested two additional hydroxamic acid–based pan-HDAC inhibitors, vorinostat and quisinostat. The IC_50_ of quisinostat (8.36 nmol/L) was similar to fimepinostat (1.19 nmol/L) but significantly lower than vorinostat (953.8 nmol/L) in the 22Rv1 cell line (Supplementary Fig. S2A). Vorinostat and quisinostat treatment of 22Rv1 cells induced a dose-dependent inhibition of AR-FL, AR-Vs, and Myc expression that coincided with hyperacetylation of histone H3 (Supplementary Fig. S2B). Together, these data suggest that HDAC inhibition rather than PI3K/AKT pathway inhibition associated with fimepinostat therapy is most likely responsible for the observed perturbation of AR and Myc levels in prostate cancer cell lines.

As AR and Myc are critical to the regulation of cell survival and proliferation in prostate cancer, we assessed the time course by which they are suppressed upon fimepinostat treatment in the 22Rv1 and LNCaP95 cell lines. Immunoblot analyses showed a clear reduction in AR protein expression by 16 hours and Myc expression by 2 hours after treatment initiation (Fig. 1D). AR inhibition in prostate cancer has been implicated in G_1_ arrest (29) while Myc has been shown to regulate the G2–M cell-cycle checkpoint—Myc overexpression diminished ionizing radiation-induced G2–M arrest in mammary epithelial cells (30) and Myc knockdown induced cyclin-dependent kinase 1 and cyclin B1–dependent G2–M arrest in Raji cells (31). Cell-cycle analysis of the 22Rv1 and LNCaP95 cell lines after 24 hours of fimepinostat treatment showed G2–M arrest (Fig. 1E) which was associated with a reduction in phospho-CDK1 levels (Fig. 1F). In addition, a reduction in cyclin B1 levels was also appreciated in the 22Rv1 but not the LNCaP 95 cell line (Fig. 1F).

As continuous dosing with HDAC and PI3K inhibitors has proven difficult in the clinic due to toxicity, we also evaluated whether intermittent dosing of fimepinostat could have relevant inhibitory effects on AR and Myc. The 22Rv1 and LNCaP95 cell lines were treated either continuously for 48 hours or intermittently (6 hours on and 18 hours off daily) for 48 hours. Immunoblot analysis showed a mild reduction of AR and Myc levels in both cell line models with intermittent treatment at doses of 30 and 100 nmol/L (Supplementary Fig. S2C and S2D).

### Suppression of AR and Myc Protein Levels by Fimepinostat is Mediated by Transcriptional Inhibition

**FIGURE 2:**
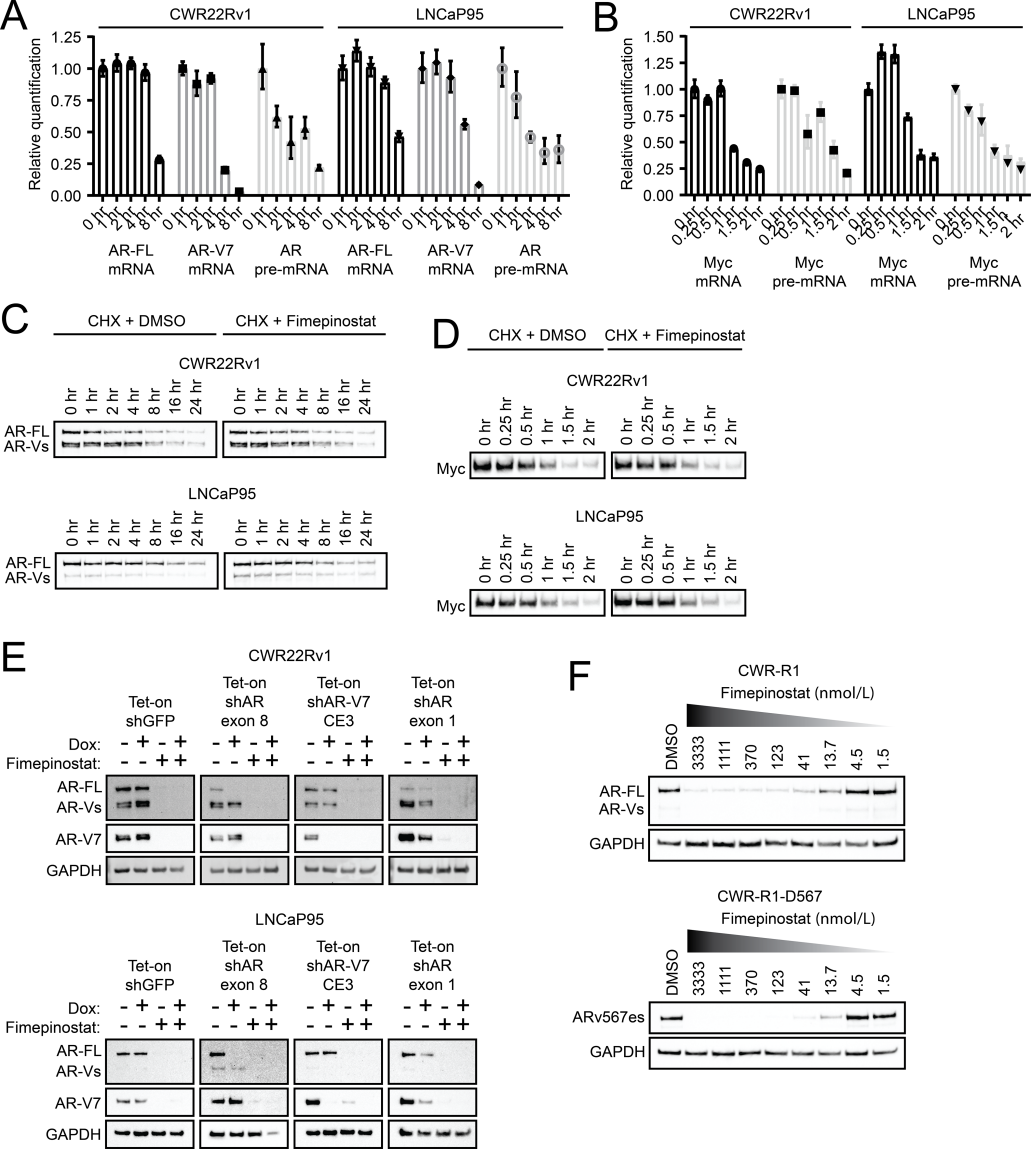
Fimepinostat inhibits AR and Myc expression at the transcriptional level and suppresses full-length and variant expression. Plots showing RQ of AR (**A**) and Myc RNA (**B**) species based on qRT-PCR analysis of 22Rv1 and LNCaP95 cells treated with 1 µmol/L fimepinostat over a time course. Results are normalized to the 0 hour condition. Immunoblot analyses of cycloheximide chase experiments to evaluate AR (**C**) and Myc protein (**D**) stability after treatment of the 22Rv1 and LNCaP95 cell lines with 0.1% DMSO or 1 µmol/L fimepinostat. **E,** Immunoblot analyses for the 22Rv1 and LNCaP95 cell lines stably transduced with conditional Tet-on shRNA expression lentiviruses (targeting GFP as a control or different AR isoforms) and treated with doxycycline and/or 1 µmol/L fimepinostat for 24 hours. **F,** Immunoblot analyses of the CWR-R1 and CWR-R1-D567 cell lines after 24 hours of treatment with DMSO control or increasing doses of fimepinostat to determine the effects on expression of AR-FL and AR-Vs including ARv567es.

We next evaluated whether the modulation of Myc and AR levels after fimepinostat treatment occurs via transcriptional or posttranscriptional mechanisms. Quantitative PCR was performed on 22Rv1 and LNCaP95 cells treated over a time course with fimepinostat using primers specifically designed to quantify AR-FL mRNA, AR-V7 mRNA, AR pre-mRNA, Myc mRNA, and Myc pre-mRNA. Our data indicated a rapid reduction in AR and Myc pre-mRNA levels beginning within 1–2 hours of fimepinostat treatment followed thereafter by a reduction in AR-FL, AR-V7, and Myc mRNA levels (Fig. 2A and B). Actinomycin D chase studies showed that fimepinostat treatment had no discernable effect on AR-FL, AR-V7, and Myc mRNA stability in the 22Rv1 and LNCaP95 cell lines (Supplementary Fig. S3A and S3B). Finally, cycloheximide chase experiments showed no clear effect of fimepinostat treatment on the protein stability of AR-FL and AR-Vs (Fig. 2C) or Myc (Fig. 2D). No quantitative change in the half-lives of AR-FL and Myc proteins in the 22Rv1 and LNCaP95 cell lines was identified after exposure to fimepinostat (Supplementary Fig. S3C and S3D). These data point to transcriptional inhibition as the major mechanism of AR and Myc suppression due to fimepinostat therapy.

On the basis of these findings, we anticipated that AR splice variant and structural variant expression should be inhibited in prostate cancer with fimepinostat treatment as the primary mode of AR inhibition is transcriptional suppression. We tested 22Rv1 and LNCaP95 cell lines that were stably engineered to express doxycycline-inducible shRNAs against GFP (control), AR exon 8, AR cryptic exon 3 (CE3) specific to AR-V7, and AR exon 1 for targeted expression of AR isoforms. In this setting, we found that fimepinostat treatment suppressed AR-FL and AR-Vs including AR-V7 (Fig. 2E). We also investigated structural variants using the CWR-R1 cell line and the subline CWR-R1-D567 in which AR exons 5–7 were deleted by genomic engineering using transcription activator-like effector nucleases (TALEN) leading to the expression of ARv567es (12). As expected, fimepinostat treatment led to reduced AR-FL levels in CWR-R1 cells and ARv567es levels in CWR-R1-D567 cells (Fig. 2F).

### Global Effects of Fimepinostat Therapy on Human Prostate Cancer

We next asked whether suppression of AR, Myc, or the PI3K/AKT pathway is critical to the antiproliferative activity of fimepinostat in prostate cancer. 22Rv1 cells engineered to stably express ectopic AR-FL, c-Myc, or constitutively active myrAKT1 that were resistant to silencing by fimepinostat demonstrated no significant change in IC_50_ in response to fimepinostat when compared with 22Rv1 control cells expressing GFP (Supplementary Fig. S4A and S4B). These data indicated that the growth inhibitory effects of fimepinostat in prostate cancer are broad and extend beyond the observed effects of fimepinostat on AR and Myc expression which was consistent with our initial observation of the broad activity of fimepinostat across prostate cancer cell lines including AR-null and NE phenotypes.

**FIGURE 3:**
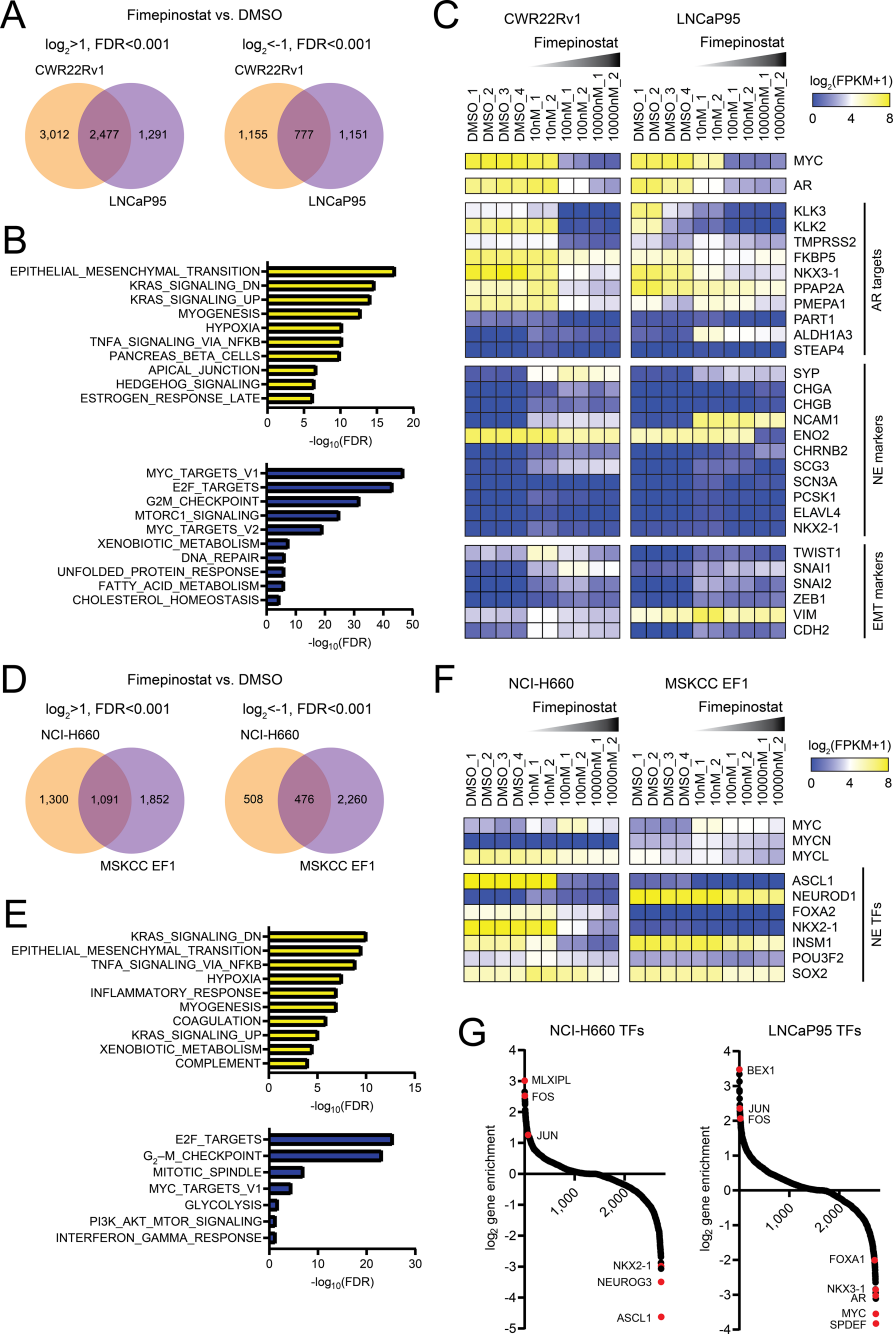
Transcriptional changes associated with fimepinostat treatment are largely conserved between CRPC and NEPC. **A,** Venn diagrams showing overlapping upregulated and downregulated genes in the 22Rv1 and LNCaP95 cell lines after treatment with 1 µmol/L fimepinostat relative to treatment with 0.1% DMSO for 24 hours. **B,** Plots showing commonly enriched (top, yellow bars) and de-enriched (bottom, blue bars) MSigDB hallmark gene sets from GSEA of overlapping genes in A. **C,** Gene expression heat map of 22Rv1 and LNCaP95 cells treated with DMSO or increasing doses of fimepinostat for 24 hours. Select genes including Myc, AR, AR targets, NE markers, and EMT markers are shown. **D,** Venn diagrams showing overlapping upregulated and downregulated genes in the NCI-H660 and MSKCC EF1 cell lines after treatment with 1 µmol/L fimepinostat relative to treatment with 0.1% DMSO for 24 hours. **E,** Plots showing commonly enriched (top, yellow bars) and de-enriched (bottom, blue bars) MSigDB hallmark gene sets from GSEA of overlapping genes in D. **F,** Gene expression heat map of NCI-H660 or MSKCC EF1 cells treated with DMSO or increasing doses of fimepinostat for 24 hours. Select genes including Myc paralogs and NE transcription factors are shown. **G,** Plot showing the overall changes in gene expression of transcription factors after treatment with 1 µmol/L fimepinostat relative to treatment with 0.1% DMSO for 24 hours. Select highly enriched and de-enriched transcription factors are labeled.

We therefore performed RNA-seq gene expression profiling to gain broader insight into the impact of fimepinostat on prostate cancer transcriptional programs. We identified 2,477 and 777 differentially expressed genes that were either coordinately upregulated or downregulated in the 22Rv1 and LNCaP95 cell lines in response to fimepinostat therapy (Fig. 3A). GSEA identified enriched ontologies associated with epithelial–mesenchymal transition (EMT), KRAS signaling, hypoxia, and TNFα signaling by NFκB (Fig. 3B). De-enriched ontologies included Myc targets, E2F targets, G2–M checkpoint, and mTORC1 signaling (Fig. 3B). We confirmed the dose-dependent suppression of AR, AR targets, and Myc gene expression with fimepinostat therapy (Fig. 3C). In addition, fimepinostat treatment was associated with the increased expression of NE markers like synaptophysin (SYP) and neural cell adhesion molecule 1 (NCAM1) as well as EMT markers such as vimentin (VIM) and snail family transcriptional repressor 1 (SNAI1; Fig. 3C).

We similarly investigated the transcriptional effects of fimepinostat therapy in NEPC cell lines. 1,091 and 476 differentially expressed genes were coordinately upregulated or downregulated in the NCI-H660 and MSKCC EF1 cell lines (Fig. 3D). GSEA revealed that enriched and de-enriched ontologies (Fig. 3E) were similar to those observed in the adenocarcinoma cell lines 22Rv1 and LNCaP95 after fimepinostat treatment. These transcriptional effects related to EMT and cell-cycle regulation have also been associated with HDAC inhibitors in other epithelial cancer types including breast cancer, pancreatic cancer, and thyroid cancer (32–34). These data appear to highlight conserved, cancer lineage–independent effects of fimepinostat therapy likely related to HDAC inhibition, which may underlie the many reports of preclinical therapeutic activity across diverse cancer types (35–39).

Focused gene expression analysis in the NEPC cell lines revealed a partial suppression of L-Myc and the NE lineage transcription factors achaete-scute homolog 1 (ASCL1) and insulinoma-associated 1 (INSM1) in the NCI-H660 cell line (Fig. 3F). However, we appreciated no significant change in Myc paralog or NE transcription factor expression in the MSKCC EF1 cell line. As NEPC and prostate adenocarcinoma are defined by distinct transcription factor networks, we evaluated the enrichment and de-enrichment of genes encoding transcription factors in the NCI-H660 and LNCaP95 cell lines after exposure to fimepinostat (Fig. 3G). Components of the activator protein (AP-1) transcription factor, Jun and Fos, were upregulated in both lines. Transcripts for ASCL1 and additional NE-associated transcription factors such as neurogenin 3 (NEUROG3) and NK2 homeobox 1 (NKX2-1) were among the most downregulated in the NCI-H660 line. In contrast, AR, NK3 homeobox 1 (NKX3-1), and forkhead box A1 (FOXA1) were significantly de-enriched in the LNCaP95 line. These findings indicate that fimepinostat can disrupt lineage-specific transcription factor expression in prostate cancer. However, we did not appreciate disruption of NEUROD1 expression in the NeuroD1-driven MSKCC EF1 NEPC line (Fig. 3F), suggesting that there may be a context- or subtype-specific effect of fimepinostat on lineage-defining transcription factors.

Notably, treatment of NCI-H660 cells with fimepinostat across a range of concentrations showed inhibition of ASCL1 protein expression at doses with on-target suppression of HDAC activity (Supplementary Fig. S5A). Similar ASCL1 inhibition with fimepinostat therapy was also confirmed in the ASCL1^+^ NEPC cell line LASCPC-01 (ref. 40; Supplementary Fig. S5A). Furthermore, treatment of the NCI-H660 cell line with the pan-HDAC inhibitors vorinostat and quisinostat also disrupted ASCL1 protein expression (Supplementary Fig. S5B). Consistent with the established opposition between the Notch pathway and ASCL1 activity in normal neuronal development and NE cancers, our RNA-seq data showed that fimepinostat treatment of NCI-H660 cells was associated with a significant reduction in ASCL1 expression and an increase in NOTCH2 and NOTCH3 expression (Supplementary Fig. S5C). We applied the selective HDAC1/2 inhibitor romidepsin to the NCI-H660 and LASCPC-01 lines and observed a decrease in ASCL1 protein expression accompanied by elevated levels of the intracellular domains of Notch2 (NICD2) and Notch 3 (NICD3; Supplementary Fig. S5D). Together, these data indicate that the pharmacologic inhibition of class I HDACs may mediate disruption of ASCL1 expression in NEPC cell lines through Notch pathway activation.

### Fimepinostat Demonstrates Potent Antitumor Activity *In Vivo* and *Ex Vivo* Across Prostate Cancer Phenotypes

**FIGURE 4:**
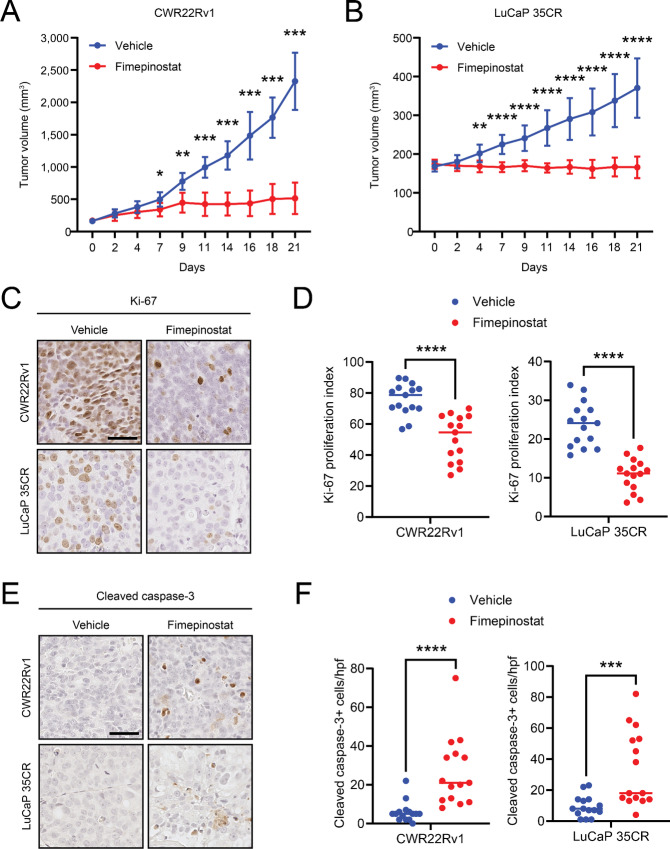
Fimepinostat treatment inhibits growth of CRPC cell line xenograft and PDX tumors. CRPC cell line xenograft tumors [(**A**) 22Rv1] and PDX tumors [(**B**) LuCaP 35CR] were treated with 75 mg/kg fimepinostat (22Rv1, *n* = 5; LuCaP 35CR, *n* = 8) or vehicle control (22Rv1, *n* = 6; LuCaP 35CR, *n* = 9) for up to 3.5 weeks. Blue = vehicle versus red = fimepinostat. *P* values = *, *P* < 0.05; **, *P* < 0.01; ***, *P* < 0.001; ****, *P* < 0.0001. Representative photomicrographs and quantification of Ki-67 (**C** and **D**) and cleaved caspase-3 (**E** and **F**) staining by IHC of 22Rv1 and LuCaP 35CR tumors treated with vehicle or fimepinostat.

We next investigated the potential for fimepinostat to suppress the in vivo growth of human prostate cancer. 22Rv1 cell line xenograft and LuCaP 35CR PDX tumors established subcutaneously in immunodeficient mice were treated with vehicle control or fimepinostat by oral gavage. The dose and treatment schedule of fimepinostat was consistent with a prior study (41) which achieved plasma levels in mice equivalent to those of the recommended phase II dose/schedule determined in a human phase I clinical trial (42). Fimepinostat treatment resulted in a statistically significant tumor growth inhibition relative to vehicle control that was first evident approximately 1 week after treatment initiation in both the 22Rv1 (Fig. 4A) and LuCaP 35CR (Fig. 4B) models. At the end of the treatment period, the LuCaP 35CR TV was 165.7 mm^3^ ± 27.7 mm^3^ with fimepinostat treatment compared with 370.3 mm^3^ ± 76.4 mm^3^ with vehicle (*P* = 0.0001). Residual tumor tissues were collected at the end of the experiment and IHC studies revealed reduced expression of the proliferative marker Ki-67 (Fig. 4C and D) and increased expression of the cell death marker cleaved caspase-3 (Fig. 4E and F) in tumors treated with fimepinostat.

We also conducted parallel experiments using ex vivo tissue slice cultures derived from 22Rv1 and LuCaP 35CR xenograft tumors. These studies revealed a reduction in the viability of 22Rv1 (28.7% ± 10.7%, P = 0.03) and LuCaP 35CR (35.1% ± 31.7%, P = 0.0014) tissue slices on day 3 with fimepinostat treatment at 300 nmol/L relative to vehicle alone (Supplementary Fig. S6A and S6B). We collected the tissue slices at the end of the study but found that those treated with fimepinostat 300 nmol/L were largely necrotic and not amenable to further evaluation. However, IHC studies of 22Rv1 and LuCaP 35CR tissue slices treated with vehicle, fimepinostat 30 nmol/L, and fimepinostat 100 nmol/L were performed to evaluate on-target effects on AR and Myc. We observed a minor but statistically significant decrease in AR expression associated with fimepinostat 100 nmol/L treatment in both the 22Rv1 and LuCaP 35CR tissue slices and fimepinostat 30 nmol/L in the LuCaP 35CR tissue slices (Supplementary Fig. S6C and S6D). Similarly, we identified a significant reduction in Myc expression associated with fimepinostat 100 nmol/L in the 22Rv1 tissue slices (Supplementary Fig. S6E and S6F).

**FIGURE 5:**
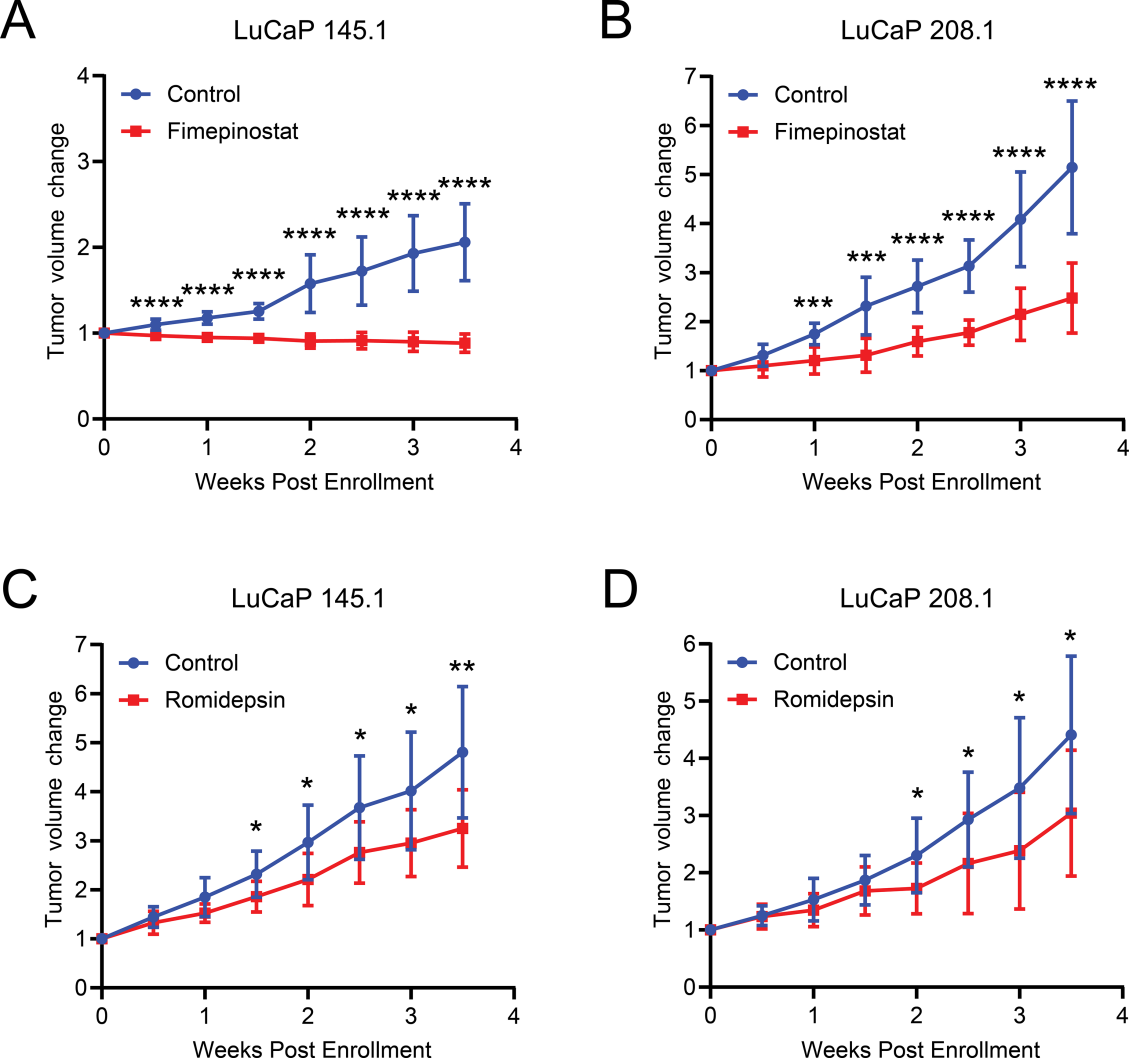
Fimepinostat significantly inhibits tumor growth in NEPC PDX models relative to the HDAC inhibitor romidepsin alone. NEPC PDX models (LuCaP 145.1 and LuCaP 208.1) were treated with 1.5 mg/kg romidepsin [(**A**) LuCaP 145.1, *n* = 11; (**B**) LuCaP 208.1 *n* = 11] or 75 mg/kg fimepinostat [(**C**) LuCaP 145.1, *n* = 9; (**D**) LuCaP 208.1 *n* = 11] or vehicle control (LuCaP 145.1, *n* = 8; LuCaP 208.1, *n* = 11) for up to 3.5 weeks. Blue = vehicle vs. red = romidepsin or fimepinostat. *P* values = *, *P* < 0.05; **, *P* < 0.01; ***, *P* < 0.001; ****, *P* < 0.0001.

We then evaluated fimepinostat therapy in mice subcutaneously engrafted with the NEPC PDX models LuCaP 145.1 and LuCaP 208.1. Significant tumor growth inhibition was observed in mice treated with fimepinostat as early as 1 week after treatment initiation (Fig. 5A and B). At the end of the treatment period, the LuCaP 145.1 TV was 145.4 mm^3^ ± 26.0 mm^3^ with fimepinostat treatment compared with 330.3 mm^3^ ± 72.2 mm^3^ with vehicle (*P* = 0.000031). Fimepinostat therapy did not induce any gross toxicities and mouse weights were not significantly different compared with vehicle treatment (Supplementary Fig. S7A and S7B).

LuCaP 145.1 tumors treated with fimepinostat demonstrated reduced Ki-67 staining and increased cleaved caspase-3 staining (Supplementary Fig. S8A). Immunoblot analysis of LuCaP 145.1 tumors (Supplementary Fig. S8B) collected after a single dose and five doses of vehicle control or fimepinostat confirmed a reduction in ASCL1 expression associated with fimepinostat treatment, as had been appreciated in *in vitro* studies. A tissue slice culture experiment was also conducted with a LuCaP 145.1 tumor and viability was diminished over time with fimepinostat 30, 100, and 300 nmol/L treatment (Supplementary Fig. S8C). IHC analysis of the LuCaP 145.1 tissue slices also showed a reduction in ASCL1 expression associated with fimepinostat 30 and 100 nmol/L treatment (Supplementary Fig. S8D and S8E).

Fimepinostat therapy was also tested in the NCI-H660 NEPC cell line xenograft model (Supplementary Fig. S8F). At the end of the treatment period, the NCI-H660 TV was 406.6 mm^3^ ± 119.6 mm3 with fimepinostat treatment compared with 1,309.1 mm^3^ ± 365.8 mm3 with vehicle (*P* = 0.00004). Collectively, these data were especially notable as these xenograft models reflect a treatment-resistant disease state for which no standard or novel therapies beyond cytotoxic chemotherapy are currently approved.

### Biologic Effects of HDAC Inhibition Across Prostate Cancer Phenotypes

As our findings indicated a broad growth inhibitory effect of HDAC inhibition in preclinical prostate cancer models, we further explored the activities of the class I-selective HDAC inhibitors etinostat and romidepsin, class I/IV-selective HDAC inhibitor mocetinostat, and the pan-HDAC inhibitor vorinostat. Romidepsin appeared the most potent as it inhibited the cell viability of both C4-2B and NCI-H660 cells at very low-nanomolar concentrations (Supplementary Fig. S9A and S9B), whereas the other HDAC inhibitors demonstrated inhibitory effects in the high-nanomolar range. When romidepsin was tested across a broader range of prostate adenocarcinoma and NEPC cell lines and dissociated tumor cells, NEPC models generally appeared more sensitive to treatment than ARPC models (Supplementary Fig. S9C). We confirmed the suppression of HDAC enzymatic activity by romidepsin, at high-picomolar to low-nanomolar concentrations in multiple prostate cancer cell lines (Supplementary Fig. S10A–S10H). These studies also showed that the NEPC cell lines NCI-H660 and LuCaP 173.1CL had lower levels of histone acetylation than the prostate adenocarcinoma cell lines C4-2B and 22Rv1 which may indicate higher levels of HDAC activity (Supplementary Fig. S10I–S1L). We also tested the antitumor activity of romidepsin in the NEPC PDX models LuCaP 145.1 and LuCaP 208.1 engrafted in mice and appreciated tumor growth inhibition (Fig. 5C and D) albeit to a lesser extent than seen with fimepinostat therapy (Fig. 5A and B). We observed a reduction in dividing cells per field in residual tumors from select prostate cancer models after treatment with romidepsin and fimepinostat, which was generally more evident in tumors with greater proliferative potential (Supplementary Fig. S11). Romidepsin therapy did not induce any gross toxicities and mouse weights were not significantly different compared with vehicle treatment (Supplementary Fig. S7C and S7D).

Pathway analysis of RNA-seq gene expression data from ARPC and NEPC cell lines and dissociated tumor cells treated with romidepsin showed enrichment in EMT, KRAS signaling, and inflammatory signaling and de-enrichment in Myc targets, E2F targets, and G2–M checkpoint (Supplementary Fig. S12A), in line with our data with fimepinostat therapy (Fig. 3B, E). NEPC models treated with romidepsin also showed an enrichment in genes associated with Notch signaling (Supplementary Fig. S12B), underscoring our prior finding that class I HDAC inhibition may activate the Notch pathway and oppose ASCL1 expression in NEPC (Supplementary Fig. S5C and S5D). A total of 104 genes were coordinately upregulated in prostate adenocarcinoma and NEPC models after treatment with romidepsin while no genes were coordinately downregulated (Supplementary Fig. S12C; Supplementary Table S1). GSEA showed enrichment of genes involved in EMT, KRAS signaling, and TNFα signaling by NFκB.

### Interaction of PI3K/AKT Inhibition and HDAC Inhibition in Prostate Cancer

**FIGURE 6:**
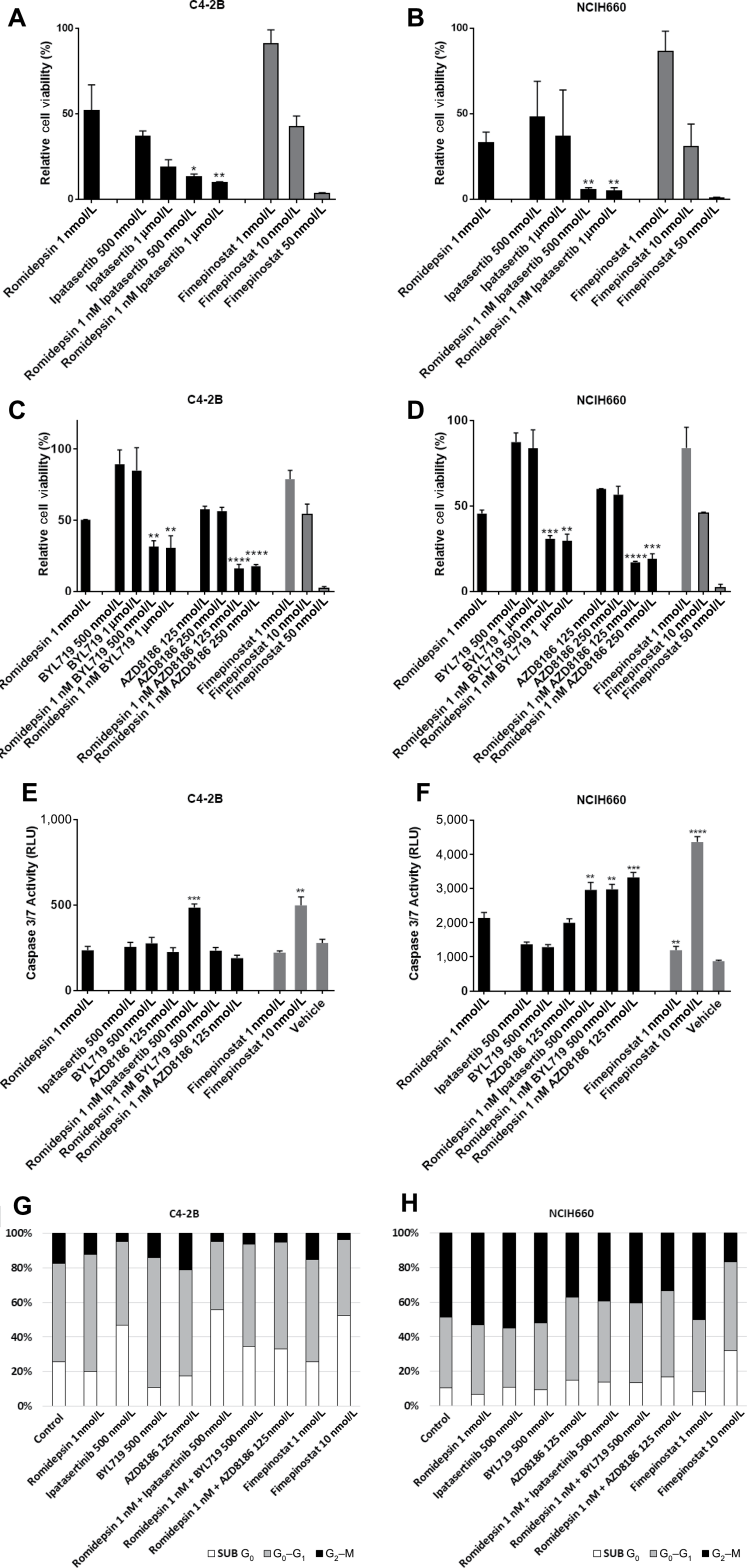
HDAC and PI3K/AKT pathway inhibition in NCI-H660 and C4-2B cells *in vitro*. Cell viability assays of C4-2B (**A**) and NCI-H660 (**B**) cells in response to romidepsin and ipatasertib combination treatment or C4-2B (**C**) and NCI-H660 (**D**) cells in response to romidepsin and BYL719 or AZD8186 combination treatment and fimepinostat for 96 hours *in vitro*. Experiments were repeated a minimum of three times. Results are expressed as percent viable cells and are normalized to vehicle-treated controls. Caspase 3/7 activity after romidepsin, ipatasertib, BYL719, or AZD8186 combination treatment of C4-2B (**E**) and NCI-H660 (**F**) cells for 48 hours *in vitro*. Cell-cycle analysis after romidepsin, ipatasertib, BYL719, or AZD8186 combination treatment of C4-2B (**G**) and NCI-H660 (**H**) cells for 48 hours *in vitro*. *P* values = *, *P* < 0.05; **, *P* < 0.01; ***, *P* < 0.001; ****, *P* < 0.0001.

We further evaluated the contribution of PI3K/AKT inhibition when combined with HDAC inhibition to the suppression of prostate cancer cell viability. We administered the AKT inhibitor ipatasertib, the selective PI3Kα inhibitor apelisib (BYL-719), or the selective PI3Kβ/δ inhibitor AZD8186 with romidepsin to treat the C4-2B and NCI-H660 cell lines. In each case, concurrent pharmacologic inhibition of PI3K/AKT and HDAC resulted in a more pronounced reduction in cell viability relative to single-agent inhibition (Fig. 6A–D). Furthermore, increased caspase 3/7 activity indicative of apoptotic cell death was observed in the C4-2B line treated with romidepsin 1 nmol/L and ipatasertib 500 nmol/L (Fig. 6E) and in the NCI-H660 with all combinations of romidepsin and ipatasertib, apelisib, or AZD8186 (Fig. 6F). Consistent with this, cell-cycle analysis by flow cytometry showed an increased sub-G0 population in the C4-2B line treated with romidepsin and ipatasertib or with fimepinostat (Fig. 6G). In contrast, combined PI3K/AKT and HDAC inhibition in the NCI-H660 line led to a slight increase in G0–G1 population and reduced G2–M population compared with controls (Fig. 6H).

To determine the nature of the interaction between PI3K/AKT and HDAC inhibition in prostate adenocarcinoma and NEPC cell line models, the cell viability of C4-2B and NCI-H660 lines were determined after treatment across a dose matrix with romidepsin and AZD8186, apelisib, or ipatasertib. On the basis of the Loewe additivity scores, romidepsin and AZD8186 demonstrated synergy while romidepsin and apelisib exhibited additivity in the C4-2B and NCI-H660 lines (Supplementary Fig. S13A, S13B, S13D, and S13E). In contrast, romidepsin and ipatasertib showed additivity in the C4-2B line and synergy in the NCI-H660 line (Supplementary Fig. S13C and S1F). These data indicate that a strategy of dual PI3K/AKT and HDAC inhibition—as with fimepinostat therapy—is likely to be superior to single inhibition due to their additive/synergistic interaction.

### Genomic Analysis of Cell and PDX Lines and Pathway Analysis of Fimepinostat and Ipatasertib + Romidepsin–treated ARPC and NEPC

PTEN encodes a dual-specificity phosphatase that functions as a direct antagonist of PI3K, a kinase involved in AKT activation. We characterized the pertinent cell lines and LuCaP PDX models and determined that none harbor PIK3CA-activating mutations (Supplementary Fig. S14). Overall, the genomic status of *PTEN, RB1, AKT1*, *PIK3CA*, and *PIK3R1* with respect to genomic alterations does not associate with fimepinostat activity in these models.

As expected fimepinostat treatment led to a disproportionate number of genes with increased expression following HDAC inhibition in the NEPC NCI-H660 cells, whereas similar numbers of genes were upregulated and downregulated in ARPC C4-2B cells which display a more open chromatin structure (Supplementary Fig. S15). We used an over-representation enrichment tool to identify gene sets in common that were over represented in fimepinostat and ipatasertib + romidepsin treated C4-2B and NCI-H660 cells relative to control (Supplementary Fig. S16; Supplementary Tables S2 and S3; ref. 17). Of the 120 upregulated genes in common between fimepinostat-treated and ipatasertib + romidepsin–treated C4-2B cells—118 were analyzed (Supplementary Fig. S16A). No significant changes in hallmark or transcription factor targets (TFT) associated gene sets were identified. In the chemical and genetic perturbations gene set the most significantly upregulated gene set was Heller_HDAC_Targets_UP (3.24 e^−8^). Of the 97 downregulated genes in common between fimepinostat and ipatasertib + romidepsin treated C4-2B cells—95 were analyzed (Supplementary Fig. S16B). The most significant downregulated hallmark pathway associated with fimepinostat and ipatasertib + romidepsin treatment was E2F_targets (2.45 e^−8^) and for TFT-associated pathways E2F_Q4_01 (4.21 e^−13^). These data verify the HDAC activity of fimepinostat and the impact on cell cycle through the downregulation of E2F-associated pathways. Of the 498 upregulated genes in common between fimepinostat and ipatasertib + romidepsin treated NCI-H660 cells—487 were analyzed (Supplementary Fig. S16C). The most significant upregulated hallmark pathway associated with fimepinostat and ipatasertib + romidepsin treatment was epithelial_mesenchymal_transition (3.82 e^−10^). For TFT-associated genes nuclear factor of activated T cells (NFAT; 9.26 e^−19^) and AP-1 (5.9 e^−14^) associated genes were significantly upregulated. The enrichment of AP-1 in the NCI-H660 cells verifies our results suggesting a role for AP-1 in fimepinostat-treated NCI-H660 cells (Fig. 3G). NFAT and AP-1 are known heterodimerization partners; Fos-Jun, cooperatively bind DNA and synergistically activate the expression of many immune-response genes in T cells (43, 44). Interestingly, a small-molecule inhibitor has been designed to target the NFAT:AP-1 transcriptional complex (45). Unlike the C4-2B cells the activation of NFAT:AP-1 would suggest the molecular response of NEPC cells to fimepinostat treatment differs from that of AR-positive adenocarcinoma. Only eight genes were downregulated both in the NCI-H660 cells treated with fimepinostat and ipatasertib + romidepsin (Supplementary Fig. S16D).

GSEA of hallmark genes from C4-2B and NCI-H660 cells treated with vehicle, fimepinostat, ipatasertib, romidepsin, or a combination of ispatasertib + romidepsin determined that all treatments lead to a significant decrease in proliferation-associated pathways, including but not limited to E2F_Targets, G_2_–M_checkpoint, MYC_targets_V1, and MYC_targets_V2 (Supplementary Fig. S16). These findings are in harmony with our studies assessing proliferation after treatment with each of the HDAC, PI3K/AKT inhibitors. In addition, as expected ipatasertib or ipatasertib + romidepsin decreased MTORC1_signaling and PI3K_AKT_MTOR_signaling pathways (Supplementary Fig. S17A). Fimepinostat and the combination of ipatasertib + romidepsin increase the Epithelial_mesenchymal_transition pathway in response to treatment. This may represent reversion to a stem-like phenotype as a mechanism of treatment resistance (46). The expression of EMT-associated genes are highlighted by the analysis of Kyoto Encyclopedia of Genes and Genomes pathways with Cell_adhesion_molecules_CAMS and ECM_receptor_interaction pathways being increased in fimepinostat and ipatasertib + romidepsin treated cells from both cell lines (Supplementary Fig. S17B).

As described above, there are common gene sets that are enriched after treatment in both the C4-2B and NCI-H660 cells in response to HDAC/PI3K/AKT inhibition. However, there are numerous differences in pathway analysis between the two cell lines underscoring the differences in the molecular pathways present and impacted in adenocarcinoma versus a NE phenotype. Taken together, these results suggest that fimepinostat and ipatasertib + romidepsin treatment has a differential effect on each cell line but share decreases in E2F target genes and cell division and increases in EMT-associated processes in response to treatment.

## Discussion

Metastatic CRPC is increasingly recognized as a heterogeneous disease entity constituting multiple molecularly distinct cancer states including prostate adenocarcinoma and NEPC that can divergently evolve and coexist within a patient and within a tumor (4, 47, 48). Therapeutic approaches directed toward a single pathway or single target such as AR antagonism or prostate-specific membrane antigen radioligand therapy may potently inhibit specific disease subtypes but are unlikely to overcome the heterogeneity commonly seen in advanced prostate cancer (49). Epigenetic therapy, including agents that inhibit histone deacetylases or DNA methylation, has yet to make an impact in the treatment of prostate cancer. A decade ago, several clinical trials evaluated HDAC inhibitors as single agents in CRPC given strong preclinical antitumor activity and evidence that HDACs regulate AR expression and signaling activity. However, the readouts from these studies were largely disappointing due to limited clinical activity and poor tolerance (50–52) with none advancing to phase III studies. Since then, investigation has focused on HDAC inhibitors with improved toxicity profiles and combining HDAC inhibitors with other antineoplastic agents.

PI3K/AKT/mTOR signaling pathway activation is highly enriched in CRPC and broadly recognized as a critical driver of prostate cancer and a key therapeutic target (53). However, strategies to inhibit TORC1/2 and PI3K in prostate cancer have largely been unsuccessful in clinical trials due to toxicity or inefficacy until recently. Data from the phase III IPAtential 150 trial in mCRPC indicate that combining the AKT inhibitor ipatasertib with abiraterone may be superior to abiraterone with placebo in extending progression-free survival (8). Whether these findings will translate into an overall survival benefit is unknown at this time. Similar to HDAC inhibition, targeting the PI3K/AKT/mTOR pathway alone may be insufficient to halt prostate cancer progression and combination approaches are likely necessary.

Fimepinostat represents one such combination therapy with a single, rationally designed molecule eliciting both pan-HDAC and PI3K inhibition (41). Fimepinostat was previously reported to demonstrate antitumor activity in prostate adenocarcinoma models through the induction of DNA damage and apoptosis (54). Our data corroborate these findings and, importantly, establish the concept that fimepinostat therapy (and combined HDAC/PI3K inhibition in general) may be therapeutically active across additional subtypes of CRPC including NEPC. A therapeutic strategy that targets phenotypically distinct prostate cancer clones would be clinically valuable given the established intratumoral and intrapatient heterogeneity of CRPC. There was no observable effect of PTEN status on fimepinostat antitumor activity in the model systems we tested (8, 55). However, our study does suggest a role for AP-1 in the molecular response to HDAC/PI3K inhibition in the NEPC phenotype. Like other HDAC inhibitors, fimepinostat blocks proliferative and cell-cycle programs in prostate cancer, independent of disease phenotype. We have also shown that fimepinostat suppresses the activities of Myc and AR, including AR variants resulting from mRNA splicing or structural alterations, by inhibiting HDACs and disrupting the transcription of these genes in prostate adenocarcinoma. Importantly, fimepinostat and other HDAC inhibitors also appear to antagonize lineage-directing ASCL1 activity in NEPC by activating Notch signaling which opposes ASCL1 expression. While much of the tumor growth inhibitory properties of fimepinostat appeared attributable to HDAC inhibition, we also demonstrate that there is additivity or synergy associated with combining HDAC and PI3K inhibitors in prostate adenocarcinoma and NEPC, demonstrating the importance of dual HDAC/PI3K targeting.

A key feature of fimepinostat is that it is an orally bioavailable drug that has demonstrated tolerability and preliminary clinical activity in a phase I trial of patients with relapsed/refractory diffuse large B-cell lymphoma with a response rate of 37% (56). Our data suggest that clinical investigation of fimepinostat or, alternatively, a combination of a selective class I HDAC inhibitor with a PI3K/AKT/mTOR pathway inhibitor such as ipatasertib should be considered for the treatment of CRPC, especially NEPC, which carries a dismal prognosis and where there is an urgent clinical need for new therapeutic development.

## Supplementary Material

Supplementary Table 1 Supplementary Table 1

Supplementary Table 2 Supplementary Table 2

Supplementary Table 3 Supplementary Table 3

Supplementary Figure 1 Supplementary Figure 1. ARPC and NEPC metastases within the same patient.

Supplementary Figure 2 Supplementary Figure 2. Effects of HDAC inhibitors on AR and Myc expression in prostate cancer cell lines.

Supplementary Figure 3 Supplementary Figure 3. Fimepinostat therapy does not impact AR or Myc mRNA and protein stability.

Supplementary Figure 4 Supplementary Figure 4. Enforced expression of AR-FL, Myc, or activated AKT1 does not rescue the growth inhibitory effects of fimepinostat therapy.

Supplementary Figure 5 Supplementary Figure 5. Fimepinostat and other HDAC inhibitors disrupt ASCL1 expression in NEPC cell lines through the activation of Notch signaling.

Supplementary Figure 6 Supplementary Figure 6. Fimepinostat treatment of prostate adenocarcinoma tissue slices causes growth inhibition and inhibition of AR and Myc.

Supplementary Figure 7 Supplementary Figure 7. Mouse body weights with fimepinostat or romidepsin therapy.

Supplementary Figure 8 Supplementary Figure 8. Fimepinostat treatment of NEPC tumors is associated with tumor growth inhibition, apoptosis, and diminished ASCL1 expression.

Supplementary Figure 9 Supplementary Figure 9. Romidepsin demonstrates broad activity across prostate cancer models with preferential potency in NEPC.

Supplementary Figure 10 Supplementary Figure 10. Histone deacetylase activity is enriched in NEPC and inhibited by romidepsin.

Supplementary Figure 11 Supplementary Figure 11. Analysis of the cell division index in viable tumor cells post-romidepsin and fimepinostat in vivo.

Supplementary Figure 12 Supplementary Figure 12. RNA-Seq and pathway analysis of romidepsin treated prostate adenocarcinoma and NEPC dissociated tumor cells and cell lines in vitro.

Supplementary Figure 13 Supplementary Figure 13. Dual AKT-PI3K and HDAC inhibition can have additive and synergistic effects in C4-2B and NCI-H660 cells.

Supplementary Figure 14 Supplementary Figure 14. Genomic assessment of RB1 and PTEN/PI3K/AKT pathway mutations in LuCaP patient-derived xenograft (PDX) models and prostate cancer cell lines.

Supplementary Figure 15 Supplementary Figure 15. RNA-seq volcano plots of C4-2B and NCI-H660 cells treated with vehicle, fimepinostat, ipatasertib, romidepsin, or a combination of ispatasertib + romidepsin.

Supplementary Figure 16 Supplementary Figure 16. Overlapping gene expression in C4-2B and NCI-H660 cells treated with vehicle, fimepinostat, ipatasertib, romidepsin, or a combination of ipatasertib + romidepsin.

Supplementary Figure 17 Supplementary Figure 17. Pathway analysis of hallmark (A) and KEGG (B) genes from C4-2B and NCI-H660 cells treated with vehicle, fimepinostat, ipatasertib, romidepsin, or a combination of ipatasertib + romidepsin.
