# Supplementary material for: Concurrent Targeting of HDAC and PI3K to Overcome Phenotypic Heterogeneity of Castration-resistant and Neuroendocrine Prostate Cancers
Source: Cancer Res Commun. 2023 Nov 20;3(11):2358–74. doi: 10.1158/2767-9764.CRC-23-0250 (PMC10658857; doi:10.1158/2767-9764.CRC-23-0250)
Supplement: Supplementary Figure 1 — ARPC and NEPC metastases within the same patient. [file crc-23-0250-s04.pdf]

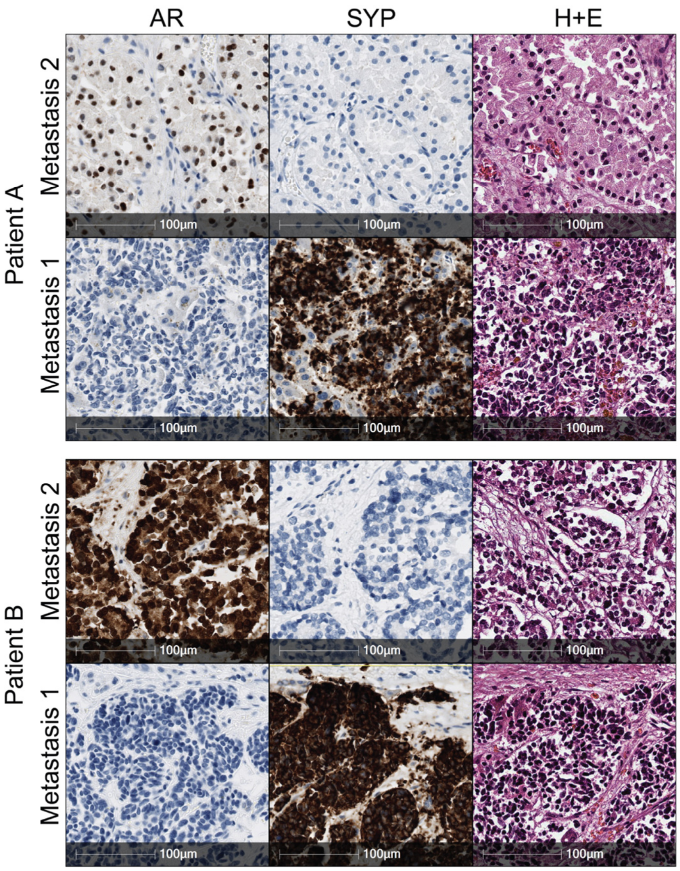

Supplementary Figure 1. ARPC and NEPC metastases within the same patient. Intertumoral expression of the androgen receptor (AR) and synaptophysin (SYP), and an H&E of two independent CRPC metastasis in each of two patients. Magnification X40.
