# Supplementary material for: Concurrent Targeting of HDAC and PI3K to Overcome Phenotypic Heterogeneity of Castration-resistant and Neuroendocrine Prostate Cancers
Source: Cancer Res Commun. 2023 Nov 20;3(11):2358–74. doi: 10.1158/2767-9764.CRC-23-0250 (PMC10658857; doi:10.1158/2767-9764.CRC-23-0250)
Supplement: Supplementary Figure 2 — Effects of HDAC inhibitors on AR and Myc expression in prostate cancer cell lines. [file crc-23-0250-s05.pdf]

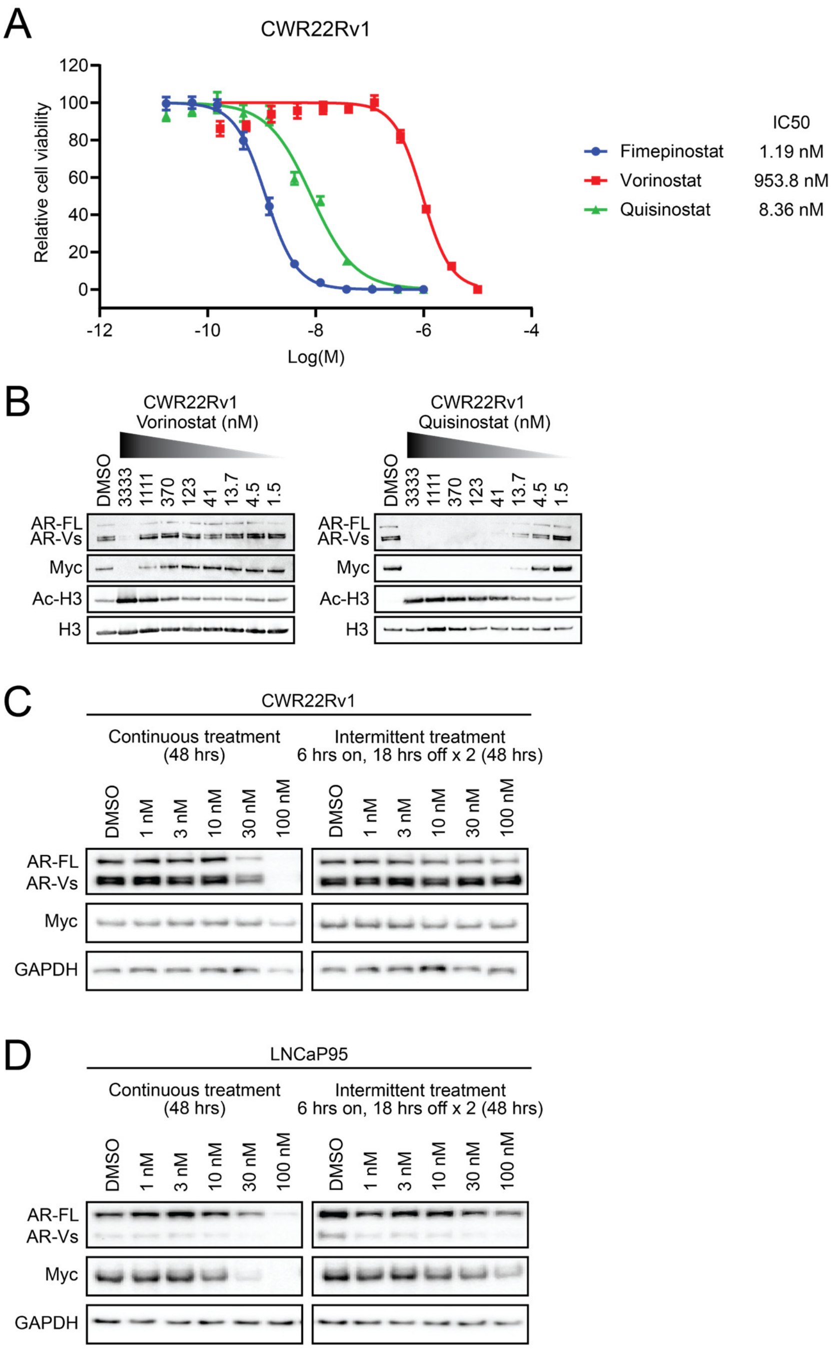

Supplementary Figure 2. Effects of HDAC inhibitors on AR and Myc expression in prostate cancer cell lines. (**A**) Dose-response curves of 22Rv1 cells treated with fimepinostat, vorinostat, or quisinostat for 96 hours. (**B**) Immunoblot analyses of 22Rv1 cells after 24 hours of treatment with DMSO control or increasing doses of vorinostat or quisinostat to evaluate for on-target activity (Ac-H3) and effects on AR and Myc. Immunoblot analyses of (**C**) 22Rv1 cells or (**D**) LNCaP95 cells after 48 hours of continuous or intermittment (6 hours on and 18 hours off daily) treatment with DMSO control or increasing doses of fimepinostat.
