# Supplementary material for: Concurrent Targeting of HDAC and PI3K to Overcome Phenotypic Heterogeneity of Castration-resistant and Neuroendocrine Prostate Cancers
Source: Cancer Res Commun. 2023 Nov 20;3(11):2358–74. doi: 10.1158/2767-9764.CRC-23-0250 (PMC10658857; doi:10.1158/2767-9764.CRC-23-0250)
Supplement: Supplementary Figure 3 — Fimepinostat therapy does not impact AR or Myc mRNA and protein stability. [file crc-23-0250-s06.pdf]

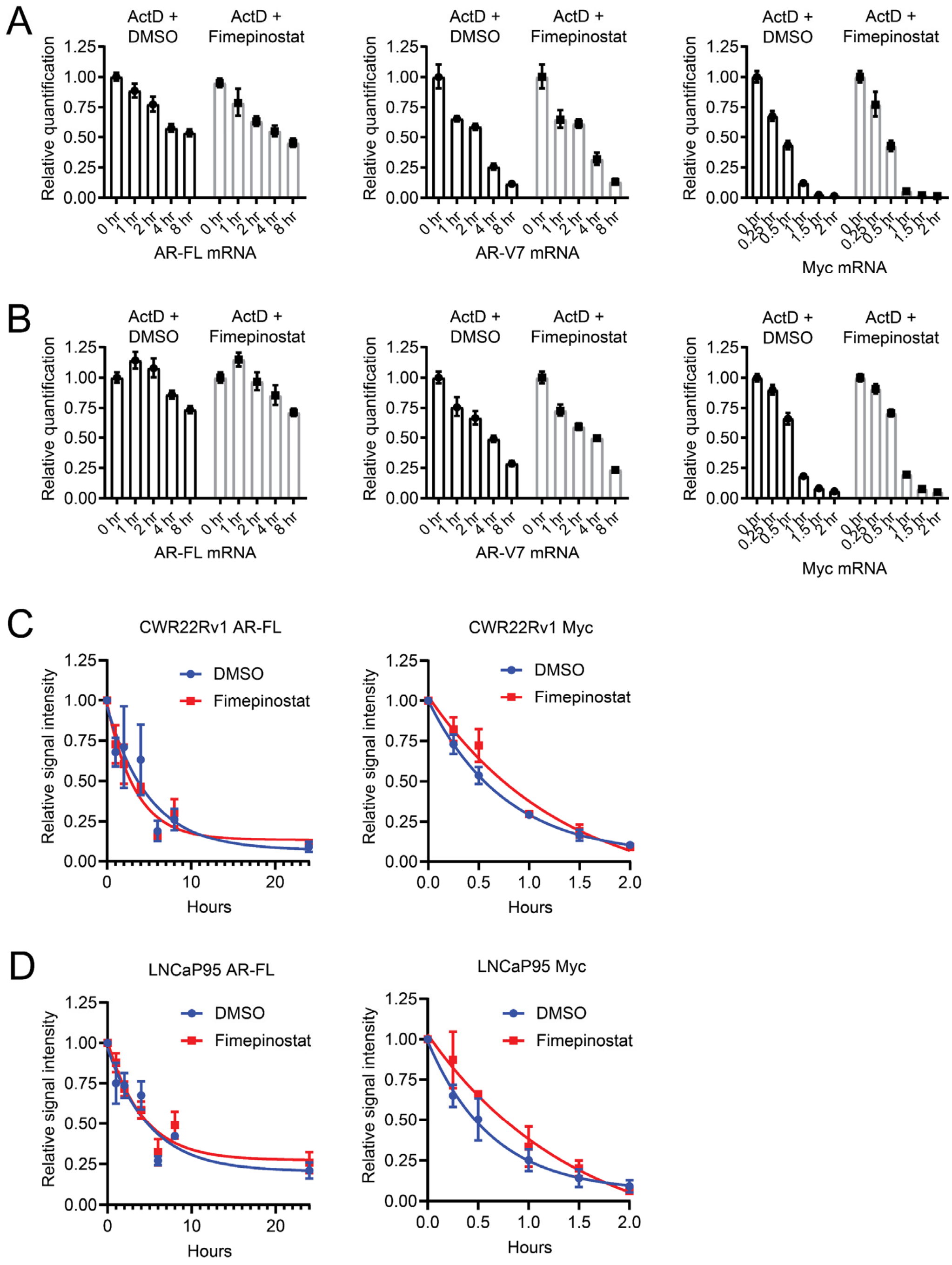

Supplementary Figure 3. Fimepinostat therapy does not impact AR or Myc mRNA and protein stability. Quantitative real time PCR analysis showing the relative stability of AR-FL, AR-V7, and Myc mRNA over time in the (**A**) 22Rv1 and (**B**) LNCaP95 cell lines after treatment with actinomycin D (ActD) and DMSO or fimepinostat 1 µM. Plots depicting the relative signal intensities of AR-FL and Myc protein bands quantified from cycloheximide chase experiments from (**C**) 22Rv1 and (**D**) LNCaP95 cell lines with DMSO or fimepinostat 1 µM treatment.
