# Supplementary material for: Concurrent Targeting of HDAC and PI3K to Overcome Phenotypic Heterogeneity of Castration-resistant and Neuroendocrine Prostate Cancers
Source: Cancer Res Commun. 2023 Nov 20;3(11):2358–74. doi: 10.1158/2767-9764.CRC-23-0250 (PMC10658857; doi:10.1158/2767-9764.CRC-23-0250)
Supplement: Supplementary Figure 4 — Enforced expression of AR-FL, Myc, or activated AKT1 does not rescue the growth inhibitory effects of fimepinostat therapy. [file crc-23-0250-s07.pdf]

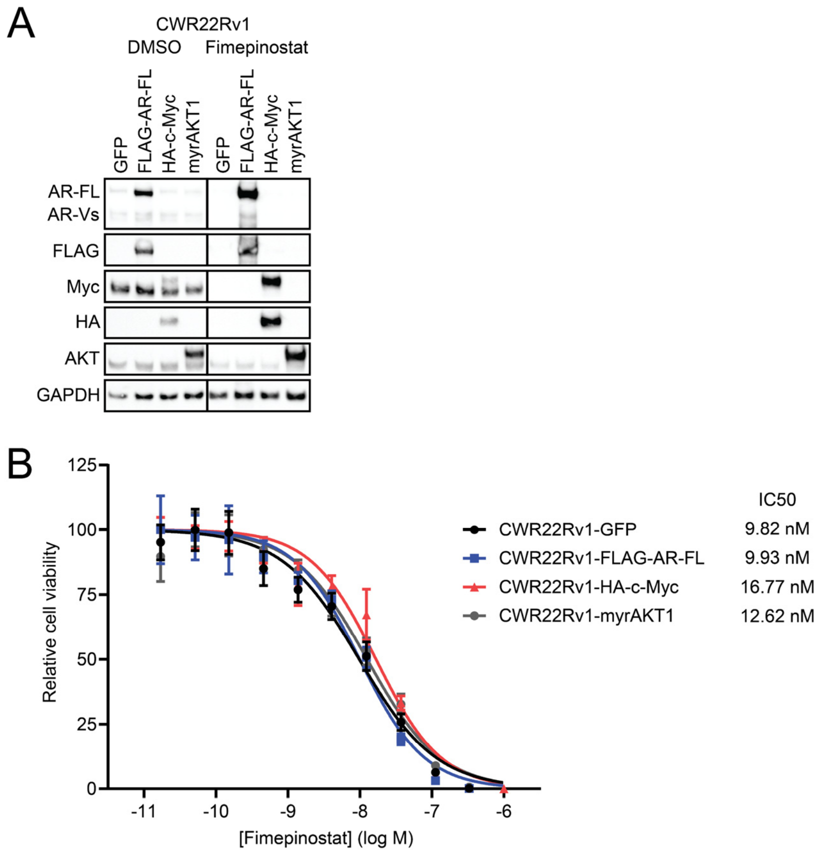

Supplementary Figure 4. Enforced expression of AR-FL, Myc, or activated AKT1 does not rescue the growth inhibitory effects of fimepinostat therapy. (**A**) Immunoblot analyses showing the stable, ectopic expression of FLAG-AR-FL, HA-c-Myc, and myrAKT1 in 22Rv1 sublines and their sustained expression after treatment with fimepinosat 1 μM for 24 hours. (**B**) Dose-response curves of 22Rv1 sublines treated with fimepinostat for 96 hours with cell viability normalized to DMSO control.
