# Supplementary material for: Concurrent Targeting of HDAC and PI3K to Overcome Phenotypic Heterogeneity of Castration-resistant and Neuroendocrine Prostate Cancers
Source: Cancer Res Commun. 2023 Nov 20;3(11):2358–74. doi: 10.1158/2767-9764.CRC-23-0250 (PMC10658857; doi:10.1158/2767-9764.CRC-23-0250)
Supplement: Supplementary Figure 5 — Fimepinostat and other HDAC inhibitors disrupt ASCL1 expression in NEPC cell lines through the activation of Notch signaling. [file crc-23-0250-s08.pdf]

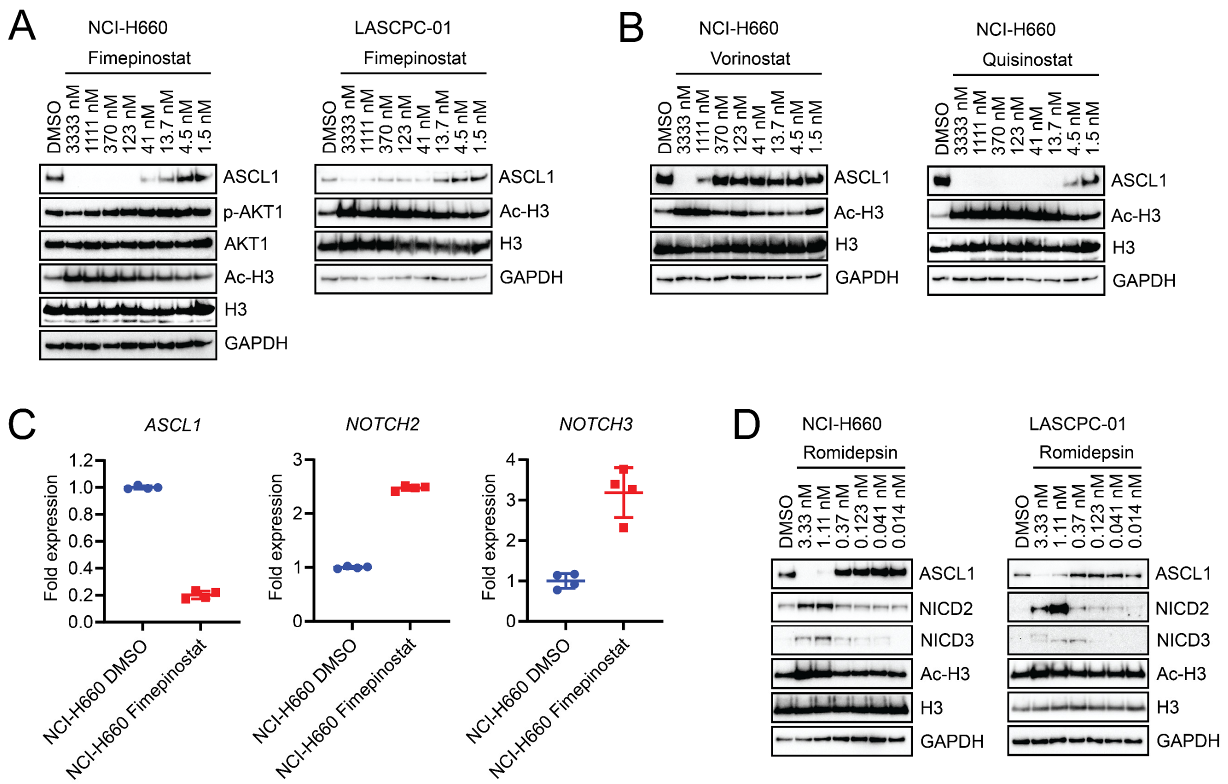

Supplementary Figure 5. Fimepinostat and other HDAC inhibitors disrupt ASCL1 expression in NEPC cell lines through the activation of Notch signaling. Immunoblot analyses showing effects on ASCL1 expression after (**A**) treatment of the NCI-H660 and NEPC cell lines with fimepinostat for 24 hours and (**B**) treatment of the NCI-H660 cell line with vorinostat and quisinostat for 24 hours. (**C**) Plots showing the relative expression of ASCL1, NOTCH2, and NOTCH3 from RNA-seq gene expression analysis of NCI-H660 cells treated with DMSO or fimepinostat 100 nM and 1 µM. (**D**) Immunoblot analyses showing effects on ASCL1, NICD2, and NICD3 expression after treatment of the NCI-H660 and LASCPC-01 cell lines with DMSO or romidepsin for 24 hours.
