# Supplementary material for: Concurrent Targeting of HDAC and PI3K to Overcome Phenotypic Heterogeneity of Castration-resistant and Neuroendocrine Prostate Cancers
Source: Cancer Res Commun. 2023 Nov 20;3(11):2358–74. doi: 10.1158/2767-9764.CRC-23-0250 (PMC10658857; doi:10.1158/2767-9764.CRC-23-0250)
Supplement: Supplementary Figure 6 — Fimepinostat treatment of prostate adenocarcinoma tissue slices causes growth inhibition and inhibition of AR and Myc. [file crc-23-0250-s09.pdf]

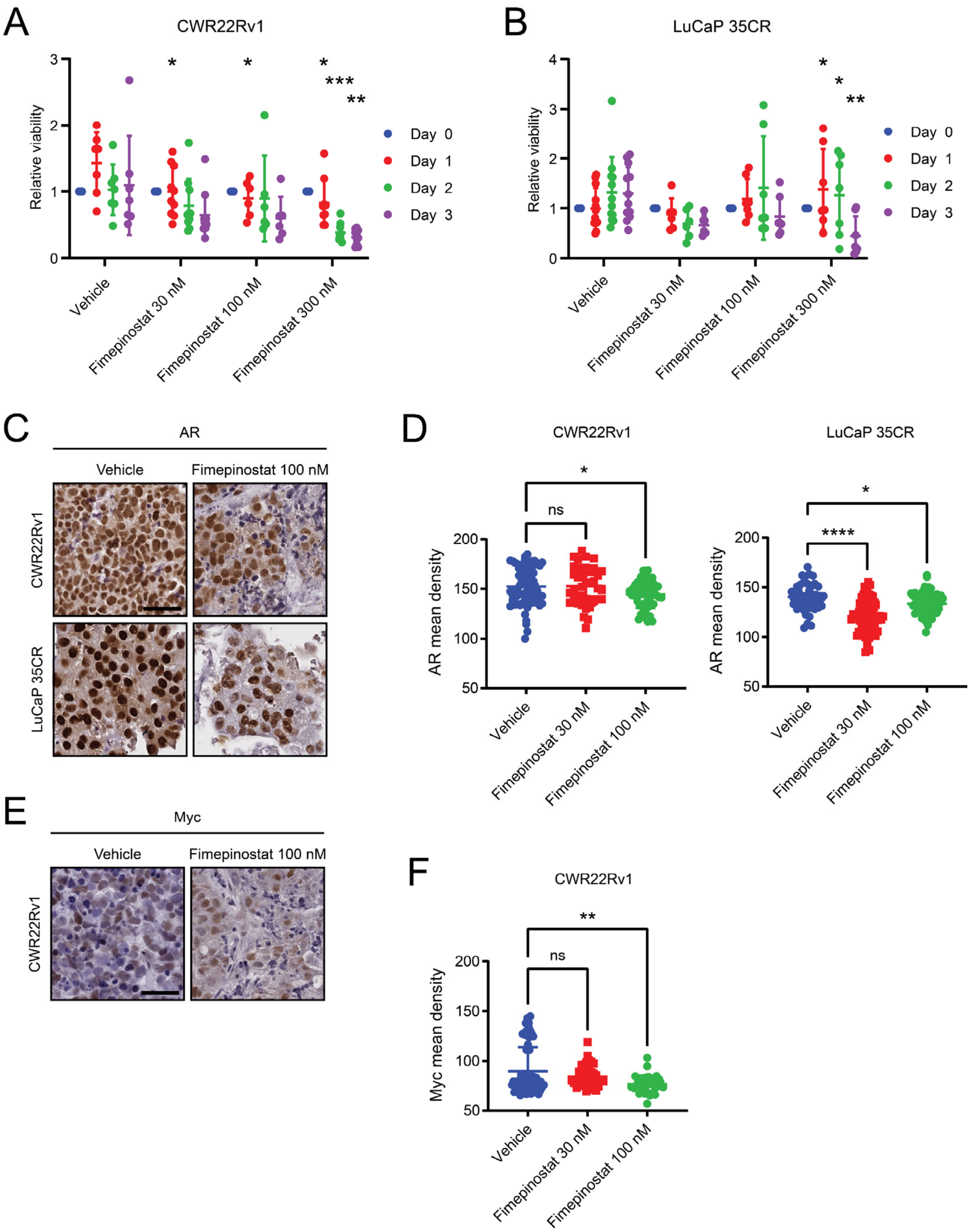

Supplementary Figure 6. Fimepinostat treatment of prostate adenocarcinoma tissue slices causes growth inhibition and inhibition of AR and Myc. Relative viability of tissue slices derived from (**A**) 22Rv1 or (**B**) LuCaP 35CR tumors is shown during short-term culture and treatment with DMSO (vehicle) or fimepinostat. (**C**) Representative photomicrographs of AR IHC on 22Rv1 and LuCaP 35CR tissue slices after treatment with vehicle or fimepinostat 100 nM for 96 hours and (**D**) quantitation of AR staining densities in 22Rv1 and LuCaP 35CR tissue slices after treatment with vehicle and fimepinostat 30 nM and 100 nM are shown. (**E**) Representative photomicrographs of Myc IHC on 22Rv1 tissue slices after treatment with vehicle or fimepinostat 100 nM for 96 hours and (**F**) quantitation of AR staining densities in 22Rv1 tissue slices after treatment with vehicle and fimepinostat 30 nM and 100 nM are shown. Scale bars represent 50 µm. P-values = *: $p < 0.05$; **: $p < 0.01$; ***: $p < 0.001$; ****: $p < 0.0001$.
