# Supplementary material for: Concurrent Targeting of HDAC and PI3K to Overcome Phenotypic Heterogeneity of Castration-resistant and Neuroendocrine Prostate Cancers
Source: Cancer Res Commun. 2023 Nov 20;3(11):2358–74. doi: 10.1158/2767-9764.CRC-23-0250 (PMC10658857; doi:10.1158/2767-9764.CRC-23-0250)
Supplement: Supplementary Figure 7 — Mouse body weights with fimepinostat or romidepsin therapy. [file crc-23-0250-s10.pdf]

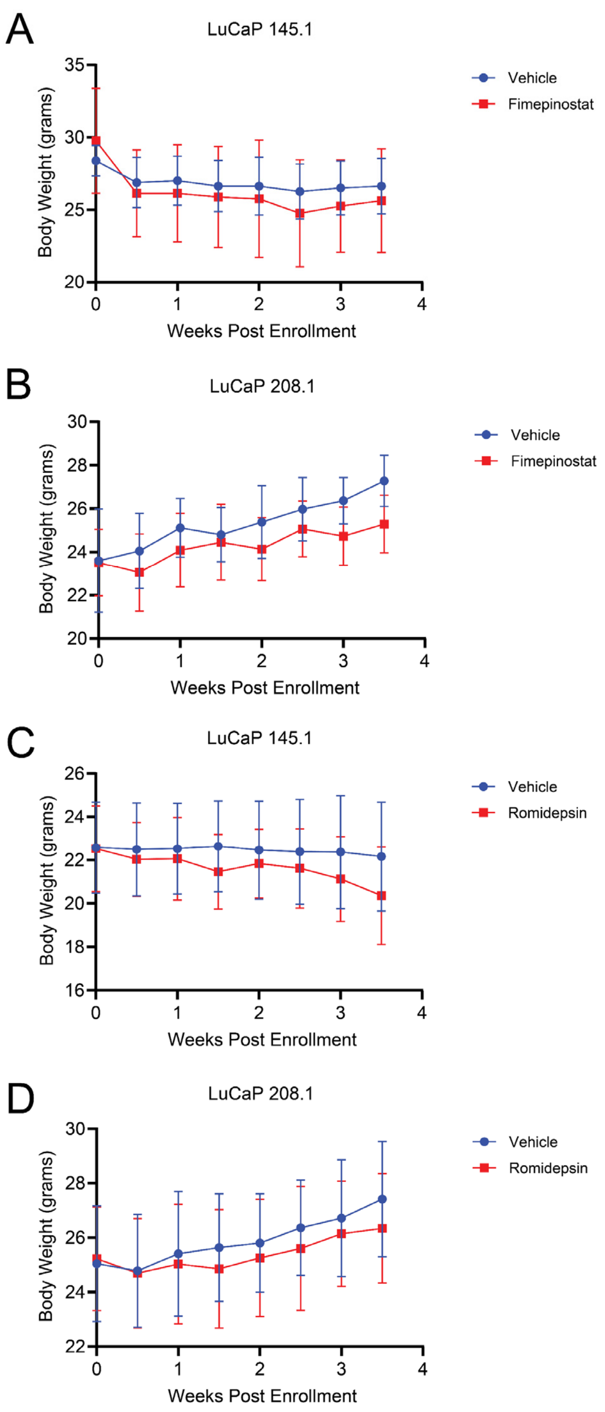

Supplementary Figure 7. Mouse body weights with fimepinostat or romidepsin therapy. Plots of the body weights of NSG mice shown in Figure 5 treated with vehicle or fimepinostat bearing (**A**) LuCaP 145.1 PDXs or (**B**) LuCaP 208.1 PDXs or treated with DMSO or romidepsin bearing (**C**) LuCaP 145.1 PDXs or (**D**) LuCaP 208.1 PDXs.
