# Supplementary material for: Concurrent Targeting of HDAC and PI3K to Overcome Phenotypic Heterogeneity of Castration-resistant and Neuroendocrine Prostate Cancers
Source: Cancer Res Commun. 2023 Nov 20;3(11):2358–74. doi: 10.1158/2767-9764.CRC-23-0250 (PMC10658857; doi:10.1158/2767-9764.CRC-23-0250)
Supplement: Supplementary Figure 8 — Fimepinostat treatment of NEPC tumors is associated with tumor growth inhibition, apoptosis, and diminished ASCL1 expression. [file crc-23-0250-s11.pdf]

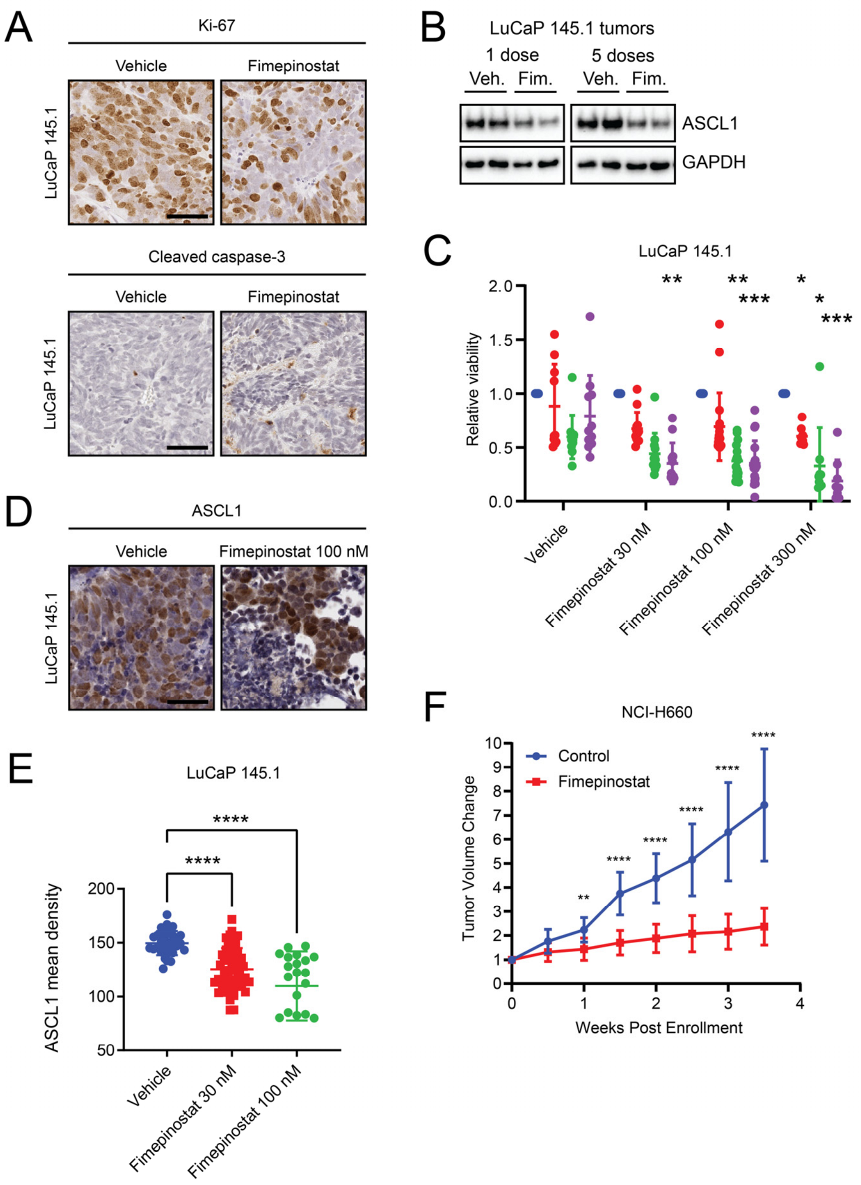

Supplementary Figure 8. Fimepinostat treatment of NEPC tumors is associated with tumor growth inhibition, apoptosis, and diminished ASCL1 expression. (**A**) Representative photomicrographs showing Ki-67 and cleaved caspase-3 IHC in residual LuCaP145.1 tumors after treatment of mice with vehicle or fimepinostat. (**B**) Immunoblot analyses of LuCaP 145.1 tumors collected after 1 dose or 5 doses of vehicle or fimepinostat and assayed for ASCL1 expression. (**C**) Relative viability of tissue slices derived from LuCaP 145.1 tumors is shown during short-term culture and treatment with DMSO (vehicle) or fimepinostat. Representative photomicrographs of ASCL1 IHC on LuCaP 145.1 tissue slices after treatment with vehicle or fimepinostat 100 nM for 96 hours and (**F**) quantitation of ASCL1 staining densities in LuCaP 145.1 tissue slices after treatment with vehicle and fimepinostat 30 nM and 100 nM are shown. Scale bars represent 50 μm. P-values = *: $p < 0.05$; **: $p < 0.01$; ***: $p < 0.001$; ****: $p < 0.0001$.
