# Supplementary material for: Concurrent Targeting of HDAC and PI3K to Overcome Phenotypic Heterogeneity of Castration-resistant and Neuroendocrine Prostate Cancers
Source: Cancer Res Commun. 2023 Nov 20;3(11):2358–74. doi: 10.1158/2767-9764.CRC-23-0250 (PMC10658857; doi:10.1158/2767-9764.CRC-23-0250)
Supplement: Supplementary Figure 9 — Romidepsin demonstrates broad activity across prostate cancer models with preferential potency in NEPC. [file crc-23-0250-s12.pdf]

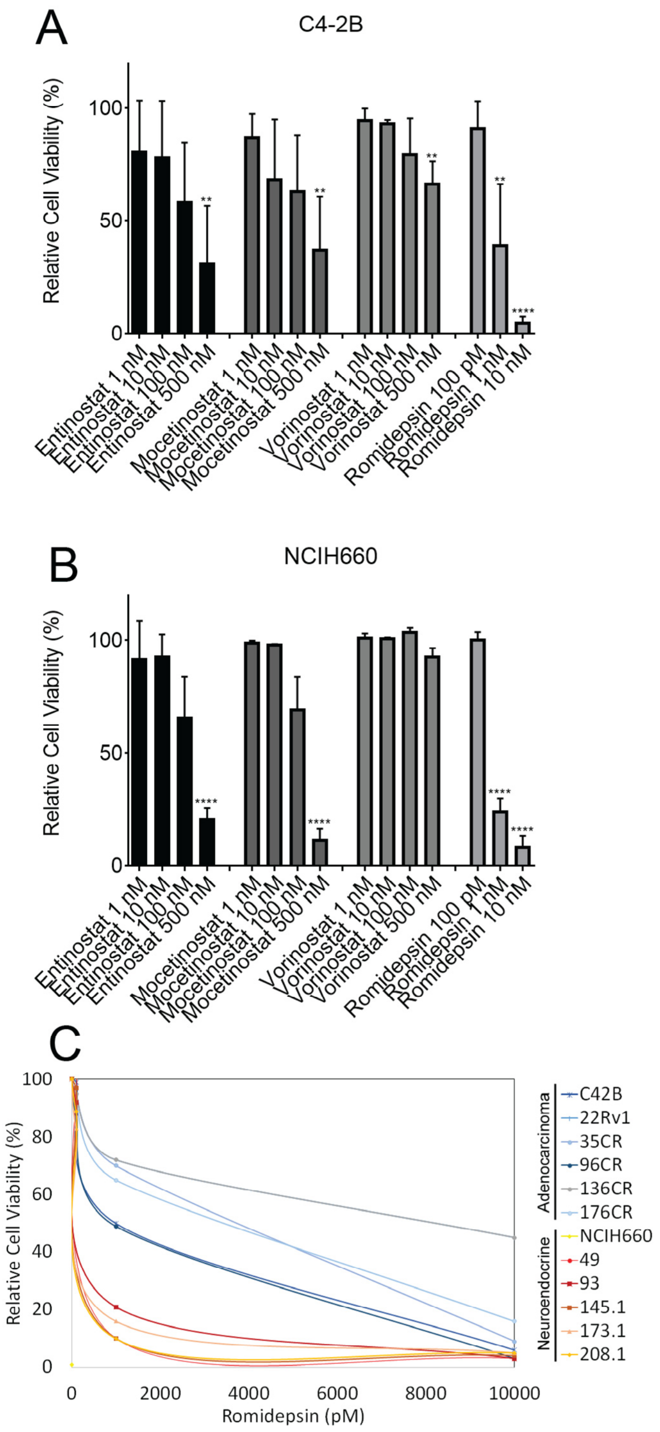

Supplementary Figure 9. Romidepsin demonstrates broad activity across prostate cancer models with preferential potency in NEPC. Cell viability assays of (**A**) C4-2B and (**B**) NCI-H660 cells in response to varying concentrations of entinostat, mocetinostat, vorinostat, and romidepsin for 96 hours *in vitro*. Results are expressed as percent viable cells and are normalized to vehicle treated controls. Experiments were repeated a minimum of three times. (**C**) A screen of dissociated LuCaP xenograft cells and cell lines (n=12) in response to increasing doses of romidepsin for 96 hours *in vitro*. P-values = *: $p < 0.05$; **: $p < 0.01$; ***: $p < 0.001$; ****: $p < 0.0001$.
