# Supplementary material for: Concurrent Targeting of HDAC and PI3K to Overcome Phenotypic Heterogeneity of Castration-resistant and Neuroendocrine Prostate Cancers
Source: Cancer Res Commun. 2023 Nov 20;3(11):2358–74. doi: 10.1158/2767-9764.CRC-23-0250 (PMC10658857; doi:10.1158/2767-9764.CRC-23-0250)
Supplement: Supplementary Figure 10 — Histone deacetylase activity is enriched in NEPC and inhibited by romidepsin. [file crc-23-0250-s13.pdf]

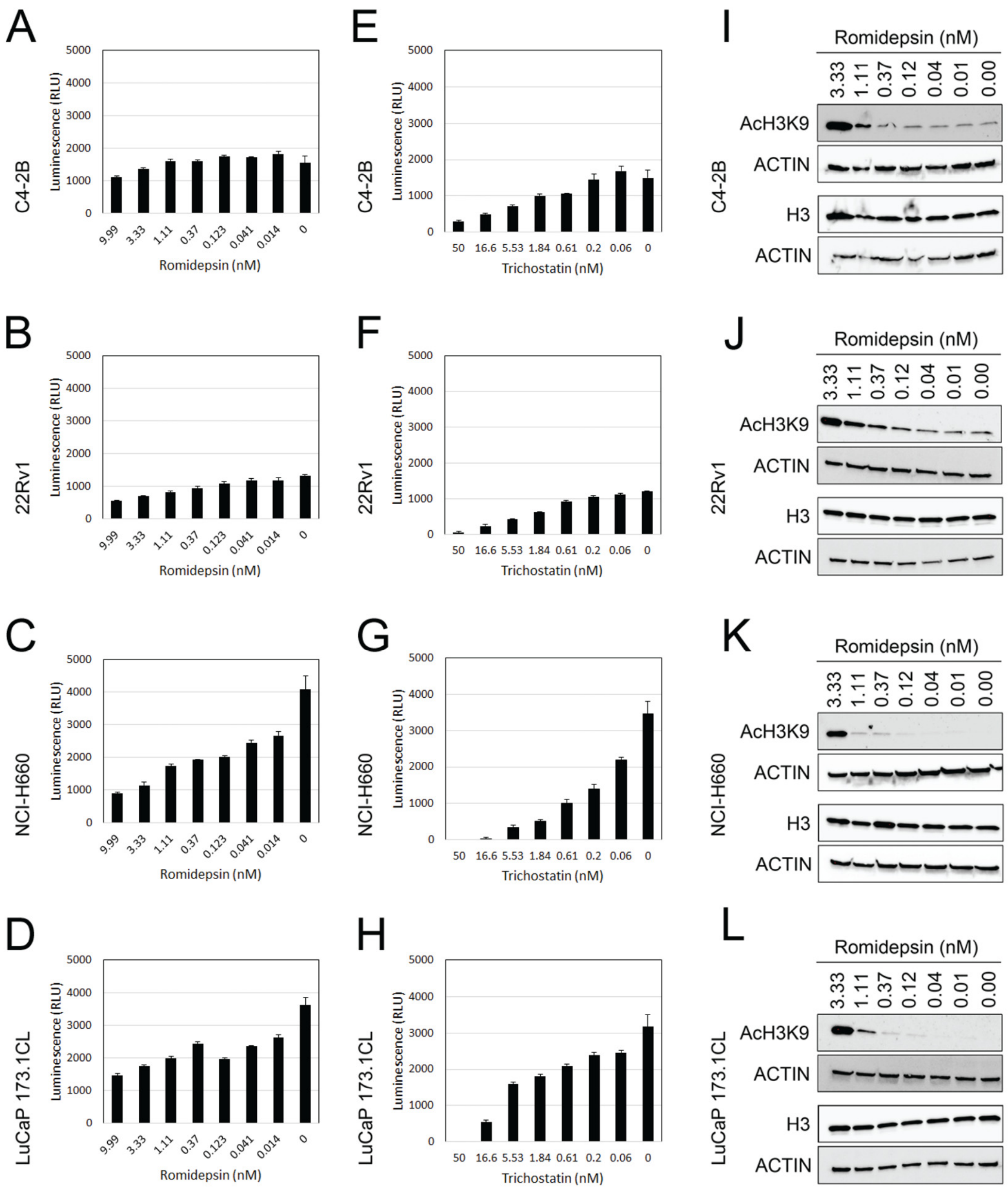

Supplementary Figure 10. Histone deacetylase activity is enriched in NEPC and inhibited by romidepsin. Histone deacetylase activity was measured in C4-2B, 22Rv1, NCI-H660 and LuCaP 173.1CL cells after treatment with increasing concentrations of romidepsin (**A**, **D**, **G**, and **J**) or trichostatin A (**B**, **E**, **H**, and **K**) for 1 hour. Levels of AcH3K9 acetylation and H3 protein expression were also assessed after treatment with increasing concentrations of romidepsin in each of the cell lines for 1 hour by immunoblot analysis (**C**, **F**, **I**, and **L**).
