# Supplementary material for: Concurrent Targeting of HDAC and PI3K to Overcome Phenotypic Heterogeneity of Castration-resistant and Neuroendocrine Prostate Cancers
Source: Cancer Res Commun. 2023 Nov 20;3(11):2358–74. doi: 10.1158/2767-9764.CRC-23-0250 (PMC10658857; doi:10.1158/2767-9764.CRC-23-0250)
Supplement: Supplementary Figure 11 — Analysis of the cell division index in viable tumor cells post-romidepsin and fimepinostat in vivo. [file crc-23-0250-s14.pdf]

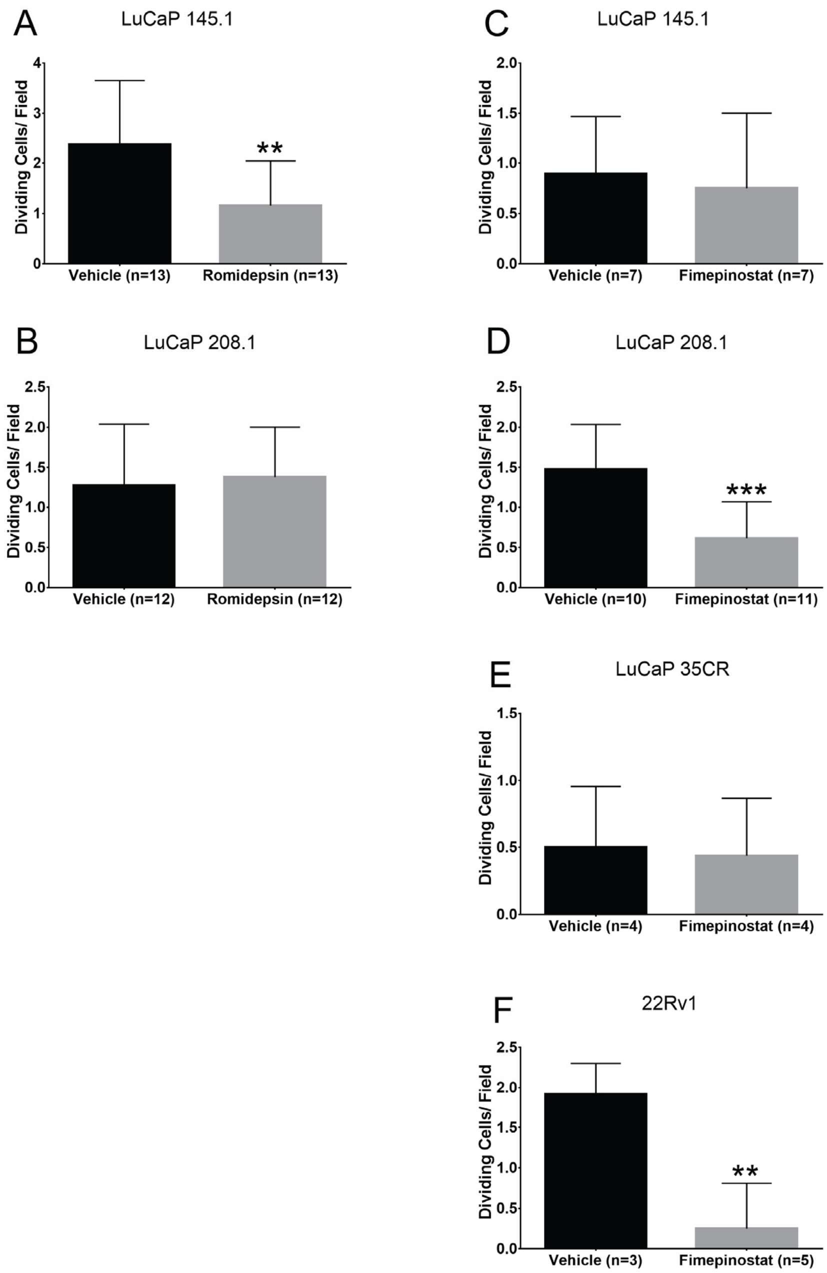

Supplementary Figure 11. Analysis of the cell division index in viable tumor cells post-romidepsin and fimepinostat *in vivo*. Cell division index in treatment resistant viable tumor in vehicle control, fimepinostat and romidepsin treated LuCaP 145.1 (**A**, **C**), LuCaP 208.1 (**B**, **D**), LuCaP 35CR (**E**), and 22Rv1 (**F**) xenografts. ** $p<0.01$, *** $p<0.001$.
