# Supplementary material for: Concurrent Targeting of HDAC and PI3K to Overcome Phenotypic Heterogeneity of Castration-resistant and Neuroendocrine Prostate Cancers
Source: Cancer Res Commun. 2023 Nov 20;3(11):2358–74. doi: 10.1158/2767-9764.CRC-23-0250 (PMC10658857; doi:10.1158/2767-9764.CRC-23-0250)
Supplement: Supplementary Figure 12 — RNA-Seq and pathway analysis of romidepsin treated prostate adenocarcinoma and NEPC dissociated tumor cells and cell lines in vitro. [file crc-23-0250-s15.pdf]

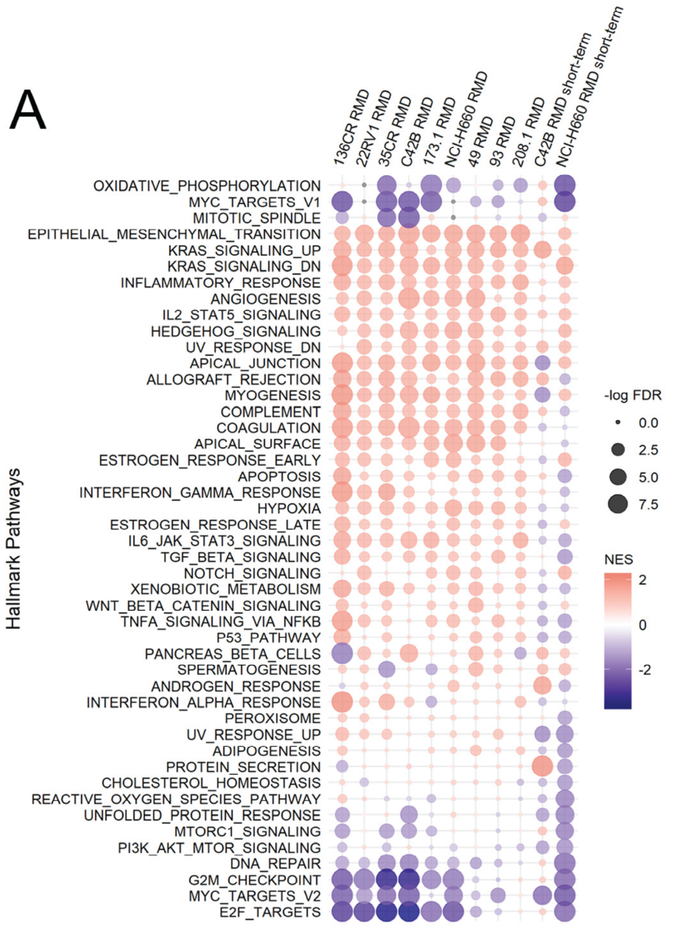

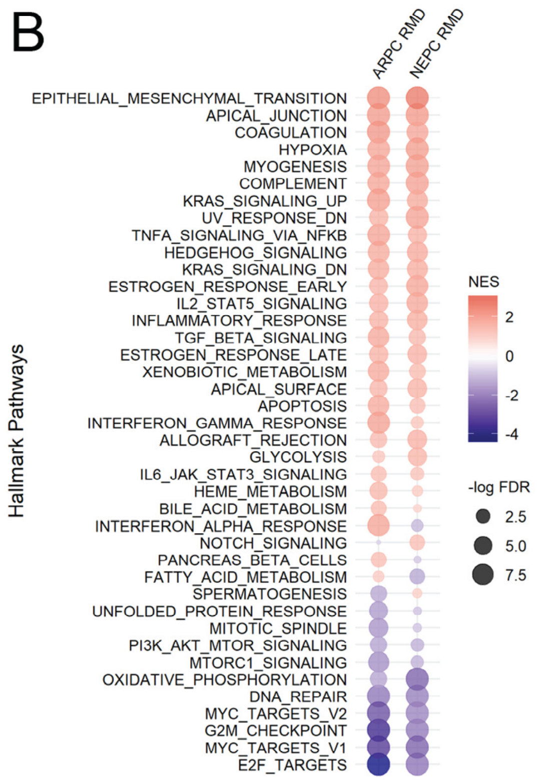

C

statistic/cutoff	ARPC RMD	NEPC RMD
all FDR<0.05	1	319
all P<0.05	2680	3544
FDR<0.05, FC>0	1	257
FDR<0.05, FC<0	0	62
P<0.05, FC>2	1046	256
P<0.05, FC<-2	279	4

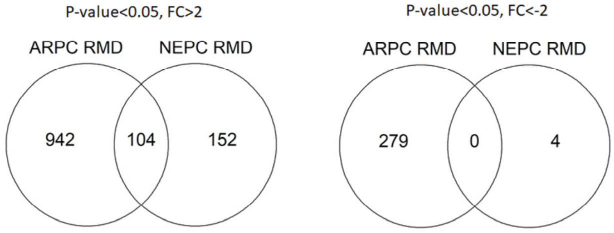

Supplementary Figure 12. RNA-Seq and pathway analysis of romidepsin treated prostate adenocarcinoma and NEPC dissociated tumor cells and cell lines *in vitro*. RNA was isolated for RNA-Seq from vehicle control (n=9) and romidepsin-treated (n=9) tumor cells. (**A**) GSEA of significantly altered genes from each line after 96 hours and 24 hours (short-term) and (**B**) each phenotype after 96 hours using the hallmark gene sets in MSigDB with FDR<0.05. (**C**) Venn diagrams of ARPC and NEPC unique and overlapping genes expressed in response to romidepsin treatment. ARPC: Androgen receptor positive prostate cancer; NEPC: Neuroendocrine prostate cancer; RMD: Romidepsin.
