# Supplementary material for: Concurrent Targeting of HDAC and PI3K to Overcome Phenotypic Heterogeneity of Castration-resistant and Neuroendocrine Prostate Cancers
Source: Cancer Res Commun. 2023 Nov 20;3(11):2358–74. doi: 10.1158/2767-9764.CRC-23-0250 (PMC10658857; doi:10.1158/2767-9764.CRC-23-0250)
Supplement: Supplementary Figure 13 — Dual AKT-PI3K and HDAC inhibition can have additive and synergistic effects in C4-2B and NCI-H660 cells. [file crc-23-0250-s16.pdf]

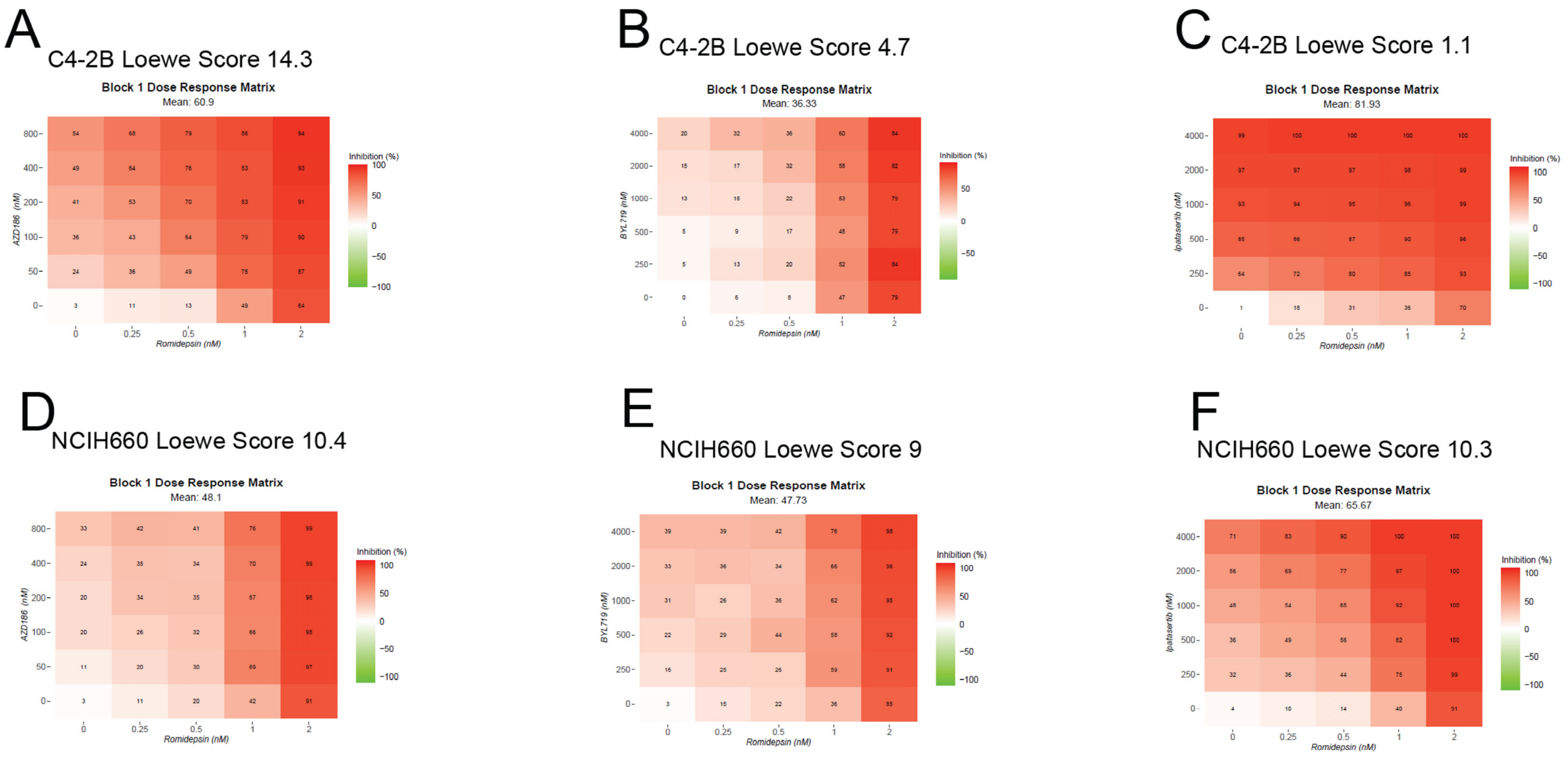

Supplementary Figure 13. Dual AKT-PI3K and HDAC inhibition can have additive and synergistic effects in C4-2B and NCI-H660 cells. Loewe analysis of C4-2B and NCI-H660 cells treated with romidepsin and (**A** and **D**) AZD186, (**B** and **E**) BYL719, and (**C** and **F**) ipatasertib. A Loewe score over 10 is considered synergistic.
