# Supplementary material for: Concurrent Targeting of HDAC and PI3K to Overcome Phenotypic Heterogeneity of Castration-resistant and Neuroendocrine Prostate Cancers
Source: Cancer Res Commun. 2023 Nov 20;3(11):2358–74. doi: 10.1158/2767-9764.CRC-23-0250 (PMC10658857; doi:10.1158/2767-9764.CRC-23-0250)
Supplement: Supplementary Figure 14 — Genomic assessment of RB1 and PTEN/PI3K/AKT pathway mutations in LuCaP patient-derived xenograft (PDX) models and prostate cancer cell lines. [file crc-23-0250-s17.pdf]

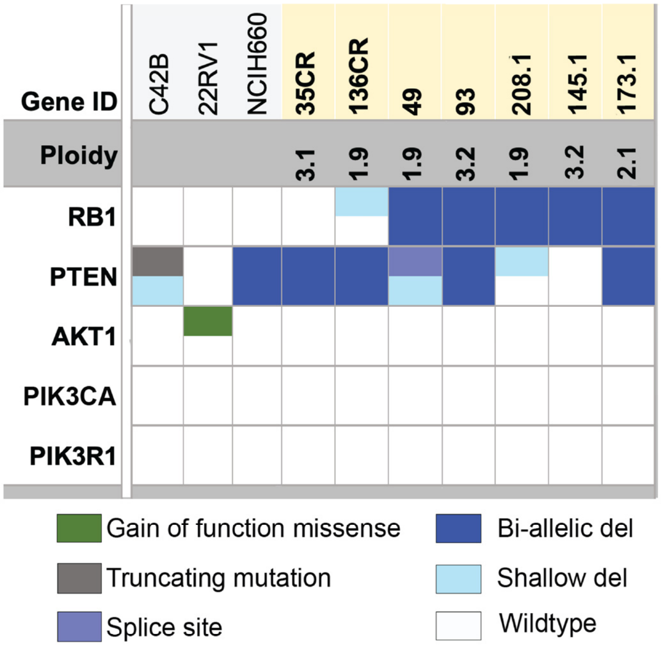

Supplementary Figure 14. Genomic assessment of RB1 and PTEN/PI3K/AKT pathway mutations in LuCaP patient-derived xenograft (PDX) models and prostate cancer cell lines.
