# Supplementary material for: Concurrent Targeting of HDAC and PI3K to Overcome Phenotypic Heterogeneity of Castration-resistant and Neuroendocrine Prostate Cancers
Source: Cancer Res Commun. 2023 Nov 20;3(11):2358–74. doi: 10.1158/2767-9764.CRC-23-0250 (PMC10658857; doi:10.1158/2767-9764.CRC-23-0250)
Supplement: Supplementary Figure 15 — RNA-seq volcano plots of C4-2B and NCI-H660 cells treated with vehicle, fimepinostat, ipatasertib, romidepsin, or a combination of ispatasertib + romidepsin. [file crc-23-0250-s18.pdf]

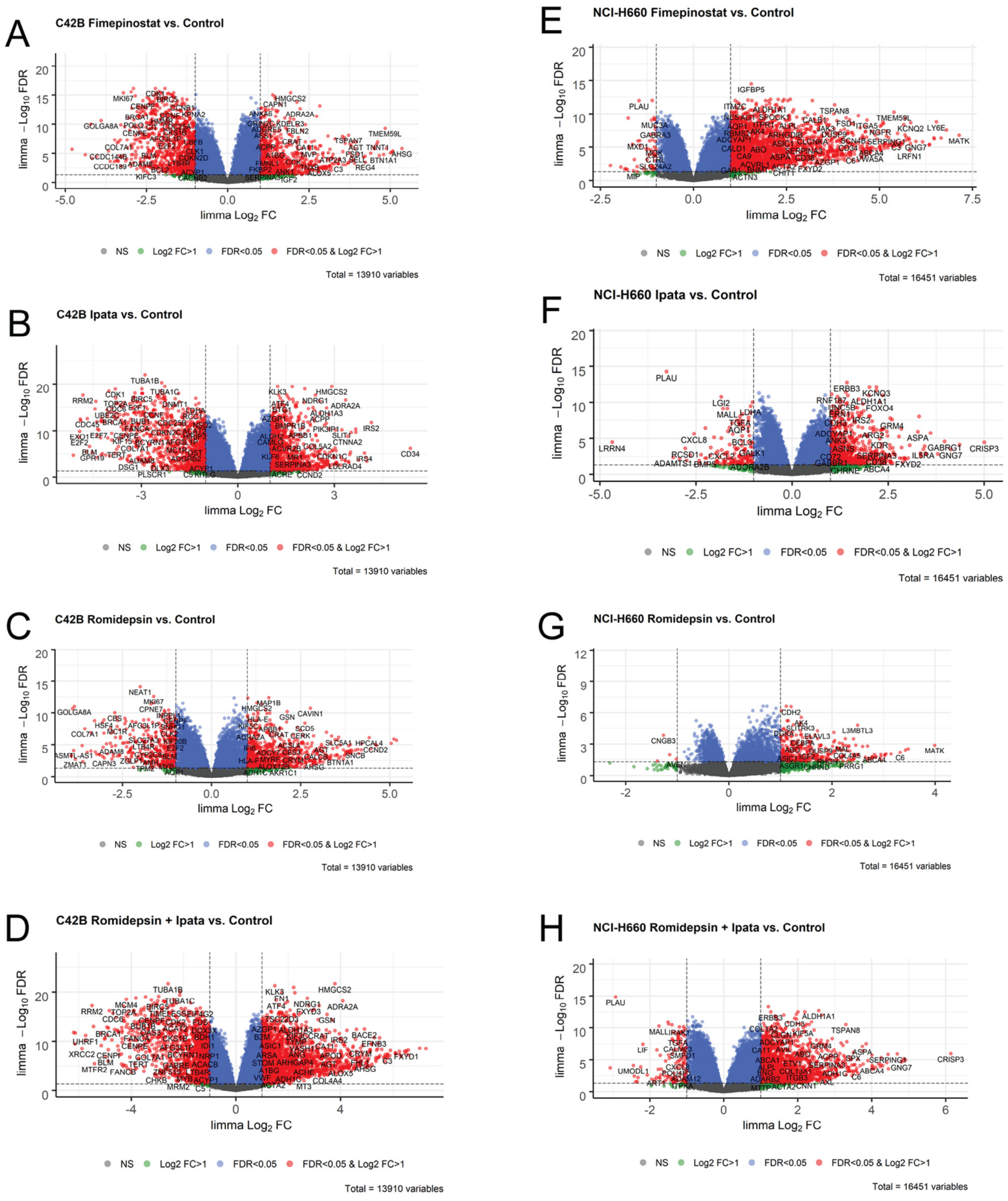

Supplementary Figure 15. RNA-seq volcano plots of C4-2B and NCI-H660 cells treated with vehicle, fimepinostat, ipatasertib, romidepsin, or a combination of ispatasertib + romidepsin. Volcano plots representing up- and down-regulated genes in response to fimepinostat, ipatasertib, romidepsin, or a combination of ispatasertib + romidepsin treatment in C4-2B (A-D) and NCI-H660 (E-H) cells. Statistically significant changes in gene expression were based on limma and only included genes with data points colored according legend; NS=not significant.
