# Supplementary material for: Concurrent Targeting of HDAC and PI3K to Overcome Phenotypic Heterogeneity of Castration-resistant and Neuroendocrine Prostate Cancers
Source: Cancer Res Commun. 2023 Nov 20;3(11):2358–74. doi: 10.1158/2767-9764.CRC-23-0250 (PMC10658857; doi:10.1158/2767-9764.CRC-23-0250)
Supplement: Supplementary Figure 16 — Overlapping gene expression in C4-2B and NCI-H660 cells treated with vehicle, fimepinostat, ipatasertib, romidepsin, or a combination of ipatasertib + romidepsin. [file crc-23-0250-s19.pdf]

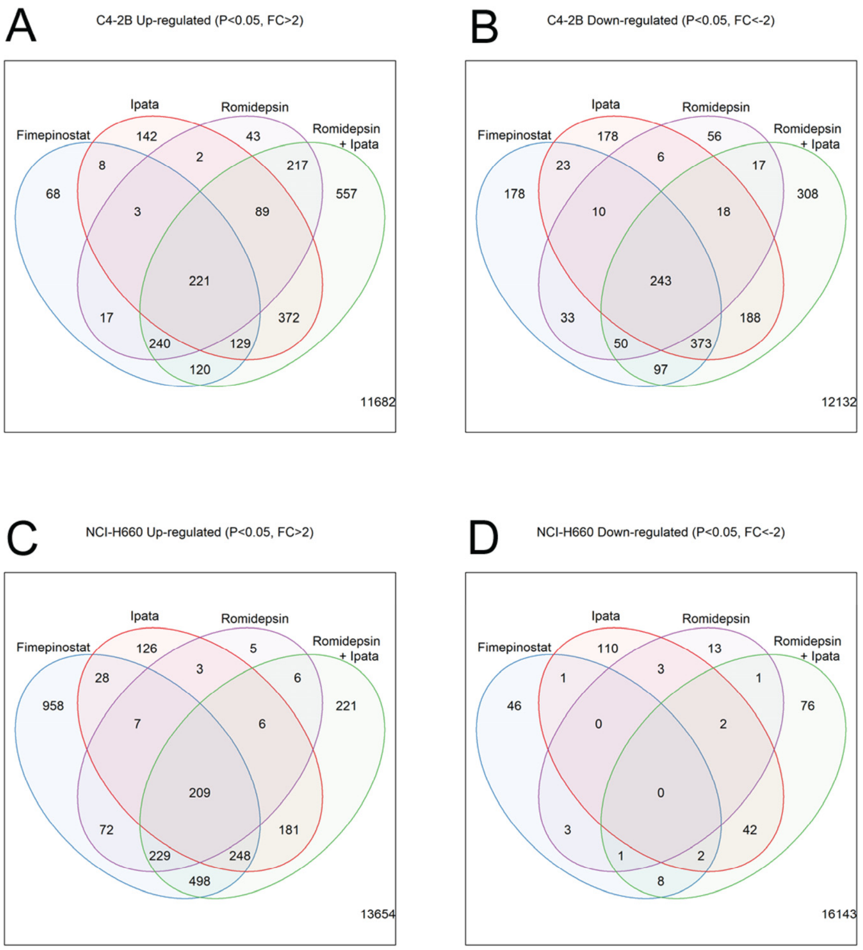

Supplementary Figure 16. Overlapping gene expression in C4-2B and NCI-H660 cells treated with vehicle, fimepinostat, ipatasertib, romidepsin, or a combination of ipatasertib + romidepsin. Venn diagram showing the overlap of significantly upregulated and downregulated genes ($p<0.05$, $FC>2$) in C4-2B cells (**A**, **B**) and NCI-H660 cells (**C**, **D**) respectively.
