# Supplementary material for: Concurrent Targeting of HDAC and PI3K to Overcome Phenotypic Heterogeneity of Castration-resistant and Neuroendocrine Prostate Cancers
Source: Cancer Res Commun. 2023 Nov 20;3(11):2358–74. doi: 10.1158/2767-9764.CRC-23-0250 (PMC10658857; doi:10.1158/2767-9764.CRC-23-0250)
Supplement: Supplementary Figure 17 — Pathway analysis of hallmark (A) and KEGG (B) genes from C4-2B and NCI-H660 cells treated with vehicle, fimepinostat, ipatasertib, romidepsin, or a combination of ipatasertib + romidepsin. [file crc-23-0250-s20.pdf]

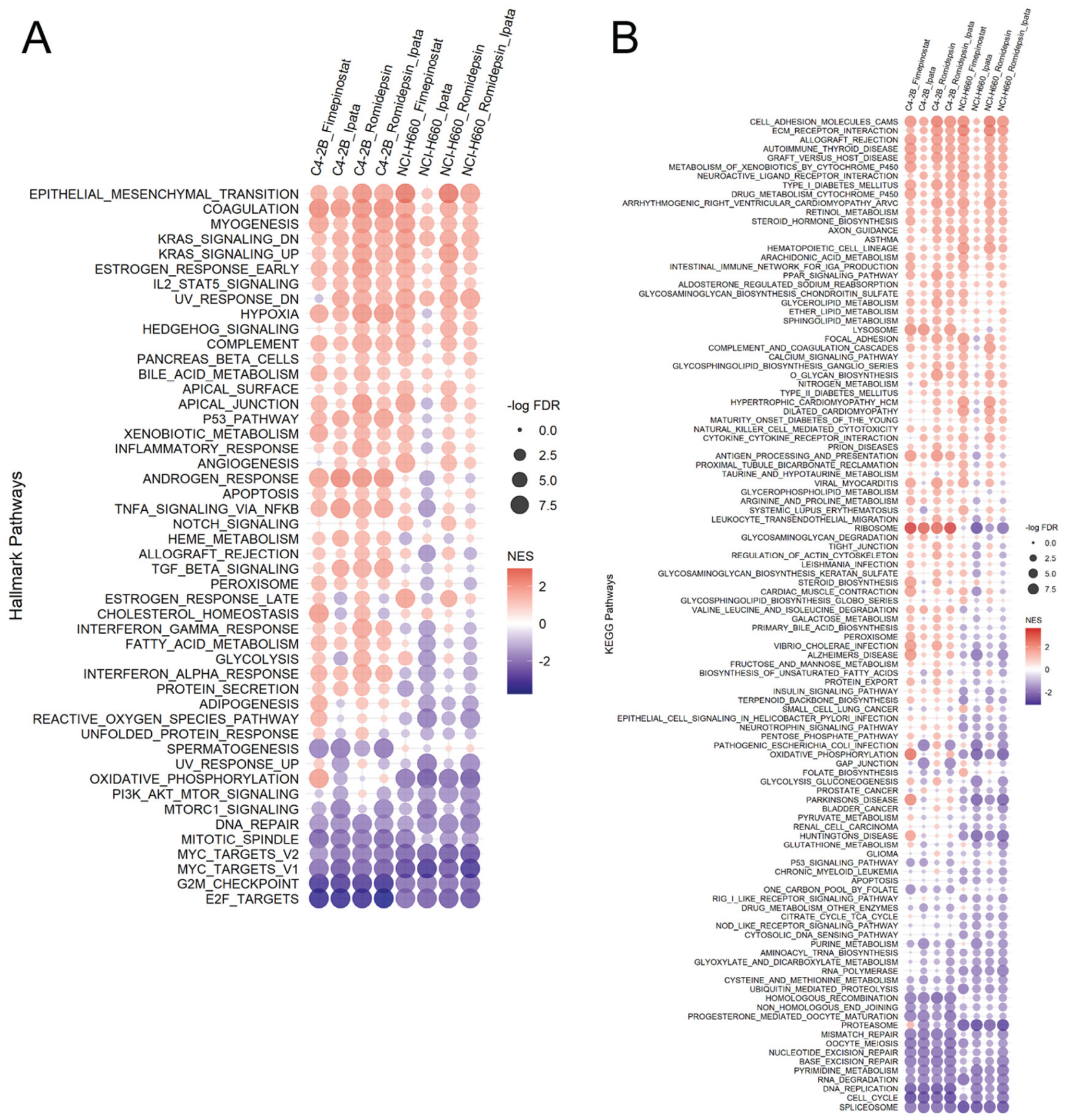

Supplementary Figure 17. Pathway analysis of hallmark (A) and KEGG (B) genes from C4-2B and NCI-H660 cells treated with vehicle, fimepinostat, ipatasertib, romidepsin, or a combination of ipatasertib + romidepsin. RNA was isolated for RNA-Seq from vehicle control, fimepinostat, ipatasertib, romidepsin, ipatasertib + romidepsin-treated tumor cells *in vitro*. (**A**) GSEA of significantly altered hallmark pathways and (**B**) KEGG pathways from each line after 24 hours in MSigDB with FDR<0.05.
